# Two cowpea Rubisco activase isoforms for crop thermotolerance

**DOI:** 10.1111/nph.70271

**Published:** 2025-06-08

**Authors:** Armida Gjindali, Rhiannon Page, Catherine J. Ashton, Ingrid Robertson, Mike T. Page, Duncan Bloemers, Peter D. Gould, Dawn Worrall, Douglas J. Orr, Elizabete Carmo‐Silva

**Affiliations:** ^1^ Lancaster Environment Centre Lancaster University Lancaster LA1 4YQ UK

**Keywords:** activase, cowpea, heat stress, photosynthesis, Rca, Rubisco, temperature response, *Vigna unguiculata*

## Abstract

- A vital crop for sub‐Saharan Africa, cowpea productivity, is threatened by climate change, including increased heatwave intensity, duration and frequency. Rubisco activase (Rca) is a key molecular chaperone that maintains Rubisco activity and, due to its thermal sensitivity, is a key target for improving crop climate resilience.
- We identified and characterised four Rca isoforms in *Vigna unguiculata* cv IT97K‐499‐35 *in vitro*: Rca1β, Rca8α, Rca10α and Rca10β. Cowpea leaf and plant growth traits were also investigated during a 5‐d +10°C heatwave that included assessment of Rubisco and Rca activity in leaf extracts and relative changes in the abundance of the four Rca isoforms.
- Cowpea Rca10α and Rca10β had higher thermal maxima, broader thermal optima and higher rates of ATP hydrolysis and Rubisco reactivation *in vitro*. In the absence of water deficit, the heatwave caused only mild effects, including increases in leaf temperature and expression of *Rca10*, small decreases in Rubisco activity and activation state and an unaltered temperature response of Rubisco activation by Rca.
- The superior Rca10α and Rca10β isoforms offer the prospect of enhancing the thermotolerance of cowpea and other crops in anticipation of more extreme future heatwaves.

## Introduction

Changes in global temperatures including heatwave events pose significant threats to global food security (Moore *et al*., 2021), making increased crop resilience against heat stress imperative (Song *et al*., 2023). Regions closer to the equator, particularly sub‐Saharan Africa, are the most vulnerable to rising temperatures (WMO, 2024). Elevated temperatures can lead to decreased photosynthesis and plant growth (Hatfield & Prueger, 2011; Bagati *et al*., 2021; Becker *et al*., 2023; Zahra *et al*., 2023). Cowpea (*Vigna unguiculata*) is a major source of protein for populations in sub‐Saharan Africa and, despite being adapted to a warm climate, it is still vulnerable to heat stress – even a 1°C increase in night temperature can result in significant yield loss (Warrag & Hall, 1984; Gonçalves *et al*., 2016; Mohammed *et al*., 2024). Therefore, it is imperative to identify physiological and biochemical traits that confer thermotolerance in cowpea to develop heat‐resilient crops (Barros *et al*., 2021).

Moderate heat stress, defined as temperatures up to 40°C (Crafts‐Brandner & Salvucci, 2000; Kumar *et al*., 2009; Ara *et al*., 2013), has a profound impact on plant photosynthesis, adversely affecting both the electron transport chain and the Calvin–Benson–Bassham (CBB) cycle (Chen *et al*., 2022). Rubisco and Chl leaf content also decline under moderate heat stress (Mathur *et al*., 2014). Chl biosynthesis enzymes exhibit susceptibility to heat stress; however, the reduction in Rubisco content cannot be attributed to denaturation of Rubisco, as alterations in secondary structure are observed only at temperatures exceeding 45°C (Li *et al*., 2002; Mathur *et al*., 2014). Increased temperatures enhance Rubisco carboxylation activity, but they also decrease Rubisco CO_2_ : O_2_ specificity and the ratio of CO_2_ : O_2_ solubilities, thereby increasing the proportion of oxygenation reactions leading to increased photorespiration (Galmés *et al*., 2016; Hermida‐Carrera *et al*., 2016). Importantly, increased carbon loss through photorespiration does not fully account for the decrease in carbon assimilation during moderate heat stress, as the Rubisco activation state has been demonstrated to decrease under both photorespiratory and nonphotorespiratory conditions during mild heat stress (Feller *et al*., 1998; Crafts‐Brandner & Law, 2000; Galmés *et al*., 2016).

Numerous studies have demonstrated that Rubisco inactivation is one of the primary causes of reduced photosynthetic efficiency during moderate heat stress (Feller *et al*., 1998; Crafts‐Brandner & Law, 2000; Portis, 2003; Ristic *et al*., 2009; Carmo‐Silva *et al*., 2015; Perdomo *et al*., 2017; Wijewardene *et al*., 2021; Scafaro *et al*., 2023). To catalyse carboxylation or oxygenation, Rubisco catalytic sites require binding of CO_2_ and Mg^2+^ as cofactors to form the active carbamylated Rubisco before binding the substrate ribulose 1,5‐biphosphate (RuBP). Rubisco can become inactivated by the binding of inhibitory sugar phosphates to carbamylated catalytic sites or by misfire products that cannot dissociate and maintain catalytic sites in their closed, inactive conformation (Orr *et al*., 2023). Inactivation can also occur when RuBP binds to an uncarbamylated Rubisco catalytic site. Inhibited Rubisco requires its molecular chaperone Rubisco activase (Rca) to restore its activity (Carmo‐Silva & Salvucci, 2013). Rca is a nuclear‐encoded protein that belongs to the AAA+ ATPases superfamily (Hazra *et al*., 2015) and, in its active multimeric form, exhibits two activities: ATP hydrolysis and Rubisco reactivation (Bhat *et al*., 2017; Serban *et al*., 2018). Rca docks on Rubisco's large subunit and, using ATP, performs conformational remodelling to release the inhibitory compound, thus modulating Rubisco activity by facilitating the dissociation of bound inhibitors. Elevated temperatures increase the frequency of Rubisco inactivation due to higher concentrations of some inhibitory substrates (Salvucci & Crafts‐Brandner, 2004). Furthermore, although Rubisco is a relatively thermostable enzyme, Rca is thermolabile and even a moderate increase in temperature results in loss of its activity (Crafts‐Brandner & Salvucci, 2000; Salvucci *et al*., 2001).

Rca isoforms within a single species vary in their regulatory properties (Zhang & Portis, 1999; Carmo‐Silva & Salvucci, 2013; Perdomo *et al*., 2019; Scafaro *et al*., 2019b). Many species express two types of Rca isoforms: longer α isoforms (43–47 kDa) and shorter β isoforms (41–43 kDa) that can be encoded by either individual genes or the same gene via alternative splicing (Carmo‐Silva *et al*., 2015). Rca α isoforms contain a C‐terminal extension with two cysteine residues shown to form a disulphide bond that is regulated by thioredoxin‐f in *Arabidopsis* (Zhang & Portis, 1999). This regulatory mechanism links Rubisco activity with alterations in the redox potential induced by environmental stimuli, such as high light (Zhang *et al*., 2002; Perdomo *et al*., 2021). The redox regulation is species‐specific, with Solanaceae species such as tobacco (*Nicotiana tabacum*) and tomato (*Solanum lycopersicum*) containing only the β Rca isoform that lacks the cysteine residues and the redox regulation of the enzyme (Carmo‐Silva & Salvucci, 2013). In species containing only redox‐insensitive Rca, light regulation appears to be mediated by changes in the ADP : ATP ratio of the chloroplast (Carmo‐Silva & Salvucci, 2013).

In addition to variation in the number of genes and the isoforms they produce, the expression and relative abundance of Rca isoforms vary among species and as a response to temperature (Law & Crafts‐Brandner, 2001; Wang *et al*., 2010; Degen *et al*., 2020; Perdomo *et al*., 2021). In rice (*Oryza sativa*), two Rca isoforms are produced via alternative splicing, and while Rca β is more abundant under optimal steady‐state conditions (Wang *et al*., 2010), heat stress induces upregulation of Rca α (Scafaro *et al*., 2016). In maize (*Zea mays*), the two isoforms derive from two genes, *ZmRcaα* and *ZmRcaβ* encoding an α (43 kDa) and a β (41 kDa) isoform, respectively (Yin *et al*., 2014). Under optimal temperature conditions, the ZmRca β protein isoform is expressed at higher levels than ZmRca α and both exhibit comparable diurnal variation in expression levels (Ristic *et al*., 2009). Jiménez *et al*. reported an increase in ZmRca α in seedlings exposed to higher temperatures; however, in more mature heat‐treated plants, this effect was not consistent (Ristic *et al*., 2009; Stainbrook *et al*., 2024). In wheat (*Triticum aestivum*), there are two *Rca* genes, one of which produces a β isoform, while the other (*TaRca2*) is alternatively spliced and produces both an α and a β isoform (Nagarajan & Gill, 2018). The Rca α : β ratio changes when wheat is exposed to heat stress, suggesting that the relative abundance of the different Rca isoforms is linked with the physiological response to heat stress (Warrag & Hall, 1984; Crafts‐Brandner & Salvucci, 2000). By contrast, in *Arabidopsis*, the Rca isoform ratio is not impacted by heat, with the α and β isoforms originating from alternative splicing continuing to be expressed at a 1 : 1 ratio (Zhang & Portis, 1999). These species‐specific responses of Rca and Rubisco regulation to heat stress illustrate the need to investigate this system directly in the crop of interest.

While the exact mechanism of Rca deactivation at high temperatures is still not fully understood, it is evident that the optimum temperature range for Rca is species‐specific and in tune with the photosynthetic optimum (Scafaro *et al*., 2016). Therefore, the temperature at which Rca loses its activity depends on environmental acclimation; that is, plants that grow at high temperatures, such as cotton and agave, maintain Rca activity at higher temperatures (Salvucci & Crafts‐Brandner, 2004; Carmo‐Silva & Salvucci, 2011; Scafaro *et al*., 2016; Shivhare & Mueller‐Cajar, 2017). Rca thermotolerance also differs between closely related species and among the native Rca isoforms within a plant species. The wild rice species *Oryza australiensis* Domin and *Oryza meridionalis* Ng. contain thermostable Rca isoforms that enable sustained high Rubisco activity and photosynthetic rate even at 45°C (Scafaro *et al*., 2016, 2018). In rice, spinach and cotton, the α isoform exhibits higher thermostability, whereas, in wheat, it is one of the two shorter β (TaRca1β) isoforms that is most thermostable (Kurek *et al*., 2007; Wang *et al*., 2010; Keown & Pearce, 2014; Degen *et al*., 2020). Increasing the thermostability of Rca is considered one of the most promising strategies for increasing Rubisco activity and enhancing photosynthesis under elevated temperatures (Carmo‐Silva *et al*., 2015; Wijewardene *et al*., 2021).

In this study, we identified four Rca isoforms in cowpea (*Vigna unguiculata*) encoded by three genes: *Rca1*, *Rca8* and *Rca10*, with the latter producing two isoforms via alternative splicing. Characterisation of their ATPase and Rubisco reactivation activities across a range of temperatures *in vitro* revealed that the two Rca10 protein isoforms have higher thermal maxima and broader thermal optima for both activities. We then examined the effect of moderate heat stress during a 5‐d heatwave at 38°C (+10°C compared with control) on the expression and abundance of Rca isoforms and subsequent impacts on Rubisco activation in the leaves of young cowpea plants. Heat treatment resulted in decreased Rubisco activation state and upregulation of *Rca8α* and *Rca10* gene expression; however, the levels of *Rca10*, which encodes both Rca10 isoforms, were consistently the lowest. Despite upregulation of the more thermotolerant isoforms, the temperature profile of Rubisco reactivation by Rca in leaf extracts (LEs) remained unaltered in heat‐treated plants. Leaf temperature increased by 6°C during the heatwave, reaching 32°C, which is within the temperature range in which Rca activity is maintained above 70% of the maximum (*T*_opt_) for all isoforms. We therefore conclude that warm‐adapted cowpea plants can cope with the mild heat stress induced by a 5‐d heatwave at 38°C in the absence of water deficit, which did not impair Rca function. Overexpression of the most thermotolerant, yet least abundant, Rca isoforms in cowpea could enable adaptation to leaf temperatures resulting from heatwaves that are more intense or accompanied by water deficit, which are likely to occur in sub‐Saharan Africa.

## Materials and Methods

### Gene identification

Cowpea (*Vigna unguiculata* L.) *Rca* genes were identified by searching the cowpea genome (Phytozome 12, cowpea genome v.1.1) using keywords and known Rca protein sequences from *Arabidopsis thaliana* (L.) Heynh., *Nicotiana tabacum* L. and *Triticum aestivum* L. (Supporting Information Table S1) (Goodstein *et al*., 2012; Lonardi *et al*., 2019). This identified three genes for *Rca* in cowpea: *Vigun01g219300* (*Rca1*), *Vigun08g150700* (*Rca8*) and *Vigun10g051600* (*Rca10*). Alignment with known sequences and key conserved features of canonical Rcas allowed identification of the products of each gene, including cases in which alternative splicing was present. Transit peptide sequences for each protein were identified by alignment to known Rca sequences (with known transit peptide locations). The analysis was repeated using the updated Phytozome 13 cowpea genome v.1.2 and yielded identical Rca isoforms (Fig. S1). Of the various transcripts for each gene, some encode the exact same protein sequence and were grouped together, some lack one or more domains of the protein that are essential for function and were disregarded, and some are not expressed and were disregarded. This analysis of transcript sequences showed that *Rca1* encodes a 384 amino acid (aa) mature protein consistent with a β isoform. *Rca8* encodes a 419 aa α isoform and a 295 aa isoform that is shorter than a regular β isoform and unlikely to be functional as it lacks part of the α‐helical and the whole C‐terminal domain, which has been shown to be essential for both ATPase and Rubisco reactivation activity (Stotz *et al*., 2011). *Rca10* encodes both a 419 aa α and a 382 aa β isoform by alternative splicing.

The cowpea *HSP20* (*Vigun06g052200*) gene, used as a heat stress marker, was identified as a homolog of the soybean (*Glycine max* (L.) Merr.) *HSP20* (*Glyma.18g094600*) shown to be upregulated in two soybean cultivars exposed to heat stress (Song *et al*., 2017). The *Arabidopsis* homolog (*At4G27670*) is also upregulated in heat stress (eFP Browser Stress Series, https://bar.utoronto.ca/) (Kilian *et al*., 2007; Winter *et al*., 2007). The *Glyma.18g094600* coding sequence was downloaded from SoyBase (https://legacy.soybase.org) and used to search the cowpea genome as described previously (Brown *et al*., 2021).

### Cloning of cowpea Rca and construction of *Escherichia coli* expression vectors

Coding regions for the mature proteins of cowpea Rca1β, Rca8α, Rca10α and Rca10β were codon optimised for *E*. *coli*, *de novo* synthesised and subcloned into pUC57 (Kan^R^) by Aruru Molecular Ltd (Dundee, UK). A Golden Gate compatible *E. coli* expression vector was created from the pET His TEV LIC plasmid (a gift from Scott Gradia, Addgene plasmid no. 29653; http://n2t.net/addgene:29653; RRID:Addgene_29653). Ligation independent cloning was used to add a Golden Gate cassette taken from pICH47742 (part of the MoClo Toolkit that was a gift from Sylvestre Marillonnet, Addgene kit # 1000000044) into pET His TEV LIC, generating the new Golden Gate compatible pET His TEV GG plasmid (Weber *et al*., 2011; Werner *et al*., 2012). Cloning a coding region into this vector allows the production of a target protein fused to an N‐terminal histidine (His) tag that can be removed with TEV protease. PCR was used to add Golden Gate overhangs to the four optimised *Rca* sequences (see Table S2, for primers used). Golden Gate reactions (final volume 15 μl) were performed with a 2 : 1 molar ratio of insert:plasmid and the following reagents (final concentration): 1× T4 DNA Ligase buffer, 0.1 mg ml^−1^ BSA, 1 mM ATP, 40 KU ml^−1^ T4 DNA Ligase and 2 KU ml^−1^ *Bsa* I. All reagents were from NEB, except ATP (Promega). Reaction conditions were 30 cycles of 37°C for 2 min followed by 16°C for 3 min, 37°C for 5 min, 80°C for 10 min and 16°C for 1 min. 5 μl of the reaction mix was used to transform *E*. *coli* DH5α cells. Resulting colonies were used to generate plasmid DNA for sequencing with primers T7F and T7R (Source Bioscience, Nottingham, UK) to verify the correct sequence including in‐frame fusion of the coding region to the His tag.

### Recombinant Rca expression and purification

Expression constructs were used to transform BL21(DE3)pLysS *E. coli* competent cells (Invitrogen). PCR and sequencing were used to confirm the presence of the correct constructs in the BL21(DE3)plysS *E. coli* cells. Several cell lines were tested for each construct to identify the cell strains with the highest expression levels for the target proteins. Expression experiments were then scaled up for protein purification.

Starter cultures (10 ml M9ZB medium, containing kanamycin and chloramphenicol, both at 50 μg ml^−1^) were inoculated from glycerol stocks and grown overnight at 37°C with 225 rpm orbital shaking. Dynamite medium (produced as described in Taylor *et al*. (2017) consisting of 12 g l^−1^ tryptone, 24 g l^−1^ yeast extract, 86 mM glycerol, 30 mM monopotassium phosphate, 72 mM dipotassium phosphate, 1.6 mM magnesium sulphate, 28 mM glucose, with added kanamycin and chloramphenicol at 50 μg ml^−1^) was seeded with the overnight starter culture (10 ml starter culture in 1 l Dynamite medium). This culture was grown for 3–4 h at 37°C with shaking at 220 rpm until OD_595_ = 0.6–0.8 and then isopropyl‐β‐D‐thiogalactopyranoside (IPTG, 0.5 mM final concentration) added to initiate protein expression. Cultures were then transferred to 24°C with 220 rpm shaking for 20 h. Cells were pelleted by centrifugation (5000 ***g***, 4°C, 20 min), washed with 40 ml buffer (50 mM HEPES pH 7.2, 300 mM NaCl and 5 mM MgCl_2_), and transferred to 50‐ml screw‐capped tubes before finally collected by centrifugation (5000 ***g***, 4°C, 20 min). The pellet was flash‐frozen in liquid nitrogen and stored at −80°C.

Cell pellets were thawed on ice and resuspended in Lysis Buffer (50 mM HEPES pH 7.2, 5 mM MgCl_2_, 300 mM NaCl, 50 mM Imidazole, 0.1% Triton X‐100 (v/v), 10 μM leupeptin and 1 mM PMSF), using 7 ml buffer for every 1 g of pellet. Cells were lysed on ice using a probe sonicator and 5 cycles of 30 s on 30 s off until the solution was homogeneous. Cell debris was removed by centrifugation (23 000 ***g***, 4°C, 30 min), and the soluble fraction was pushed through a 0.45‐μm syringe filter before chromatography.

Purification of the Rca protein was achieved using an Next‐Generation Chromatography (NGC) chromatography system (Bio‐Rad), equipped with a nickel‐charged immobilized metal affinity chromatography (IMAC) column (1 ml HisTrap Fast Flow; Cytiva, Amersham, UK) and a desalting column (10 ml Bio‐Scale Mini Bio‐Gel P6 Desalting Cartridge; Bio‐Rad). IMAC and desalting columns were equilibrated with Equilibration Buffer (5 ml: 50 mM HEPES pH 8, 300 mM NaCl, 5 mM MgCl_2_ and 50 mM imidazole) and Final Buffer (13 ml: 50 mM HEPES pH 8, 5 mM NaCl and 5% glycerol), respectively. The sample was applied to the IMAC column, followed by Wash Buffer (15 ml: 50 mM HEPES pH 8, 300 mM NaCl, 5 mM MgCl_2_ and 125 mM imidazole). Bound protein was eluted using a higher concentration of imidazole (2.1 ml: 50 mM HEPES pH 8, 300 mM NaCl, 5 mM MgCl_2_ and 300 mM imidazole) and directly applied to the desalting column equilibrated in Final Buffer (50 mM HEPES pH 8, 5 mM MgCl_2_ and 5% glycerol). Fractions (1 ml) containing protein were identified using a UV/Vis detector reading at 280 nm. Fractions containing the highest protein concentration (determined using Bradford reagent, Bio‐Rad) were pooled, ATP and DTT (0.2 mM and 5 mM final concentration respectively) added, and 50–100 μl aliquots frozen in liquid nitrogen before storage at −80°C.

### *In vitro* characterisation of Rca thermal optima

The rate of ATP hydrolysis by Rca isoforms was assayed using the method described by Chifflet *et al*. (1988), with modifications as described within Degen *et al*. (2020). ATP hydrolysis was measured at a range of temperatures (20–55°C, with 5°C increments) using 5‐min assays, with reactions initiated through the addition of Rca (final concentration 1‐μM Rca monomer) and quenched via the addition of sodium dodecyl sulfate (SDS). After quenching, all reactions and standards were kept at 4°C until inorganic phosphate (P_i_) determination. The P_i_ released during ATP hydrolysis was determined by measuring the absorbance of molybdenum blue at 850 nm in a spectrophotometer (SpectroStar Nano; BMG Labtech, Aylesbury, UK).

Rubisco reactivation assays were conducted at a range of temperatures (20–45°C, with 5°C increments) following the procedures of Barta *et al*. (2011) and Degen *et al*. (2020). Briefly, Rubisco purified as per Amaral *et al*. (2024b) was used to prepare inhibited Rubisco complexes (ER) through spin‐desalting purified Rubisco in CO_2‐_ and Mg^2+^‐free buffer followed by incubation with RuBP overnight at 4°C (Barta *et al*., 2011). The two‐stage reactivation assay comprises a first stage assay where Rca is added to inhibited Rubisco (ER) at a test temperature (20–45°C, with 5°C increments), with aliquots from this assay taken at time intervals to initiate the second stage assay that measures Rubisco activity. The Rubisco activity measured at each time point is a function of the Rca, time allowed for reactivation and the temperature of the first stage (Fig. S2). The first stage assays contained 5 mg ml^−1^ Rubisco, 1 mg ml^−1^ Rca and 5 mM ATP. With known quantities of Rca and Rubisco and appropriate controls, Rca reactivation of Rubisco can be established following the calculations described within Degen *et al*. (2020). Maximum Rubisco activity was determined at every temperature and was comparable among assays with each of the four Rca isoforms.

Independent purifications of each isoform were used as biological replicates. For ATPase activity, three biological replicates were measured for Rca8α and four for each of the rest of the isoforms. For Rubisco reactivation, four biological replicates were measured for temperatures 20, 25, 30, 32°C and three for temperatures 38, 40, 45°C. To estimate maximum ATP hydrolysis and Rubisco activation activities and the corresponding temperatures at which the maximum values are attained, second‐ to third‐order polynomials and generalised additive models (GAM) were fitted to the experimental data using the gam function from the mgcv 1.8‐24 package in R (Wood, 2017). The model that best fit the experimental data was selected based on the Akaike information criterion (AIC) using the AIC function (Akaike, 1974) (Table S3). The selected model was fitted to each set of the biological replicates as well as the whole dataset (Table S4). *T*_max_ was calculated as the temperature corresponding to maximum activity. *T*_opt_ was calculated as the range in which activity was maintained above 70% of maximum value. *T*_0.5_ was calculated as the temperature in which activity dropped below 50% of maximum value.

### Plant growth and sampling

*Vigna unguiculata* cv IT97K‐499‐35 seeds (kindly provided by B.B. Singh) were germinated in 0.6‐l Deepots (D40H; Stuewe & Sons, Tangent, OR, USA) containing a 1 : 1 (v/v) mixture of nutrient‐rich compost (Petersfield Growing Mediums) and silver sand (horticultural grade, Royal Horticultural Society). Plants were grown in eight growth cabinets (MC1000; Snijders Labs, Tilburg, the Netherlands) and watered as needed to soil saturation. Cabinets were maintained at day : night temperatures of 28°C : 18°C, 70% relative humidity and 400‐ppm CO_2_ concentration (control conditions). A 12‐h photoperiod of constant light of 850 μmol m^−2^ s^−1^, canopy level was maintained via LED lighting (NS1 spectrum; Valoya, Helsinki, Finland). Four cabinets were randomly selected to apply a heat treatment starting at 13 d after sowing (das) including an intermediate day with day : night temperatures of 34°C : 24°C followed by 5 d of 38°C : 28°C (Fig. S3a). Data loggers (OM‐EL_USB‐2; Omega Engineering Ltd, Manchester, UK) were placed at canopy level in each cabinet to record temperature and relative humidity every 15 min (Fig. S3b).

Twenty cowpea plants were grown in each cabinet and sampled or measured for different analyses following a randomised design (Fig. S4). All samples and measurements were taken from the central leaflet of the first trifoliate leaf. Leaf disc samples were taken 4–5 h into the light period, after which plants were cut above the unifoliate leaves to reduce shading of neighbouring plants. Separate plants were used for sample collection in subsequent time points. For nondestructive measurements, such as plant growth traits and Chl content, the same individuals were measured at all time points.

Growth measurements were obtained at 15, 17 and 22 das (Days 2 and 4 of the heatwave and on Day 4 of the postheatwave recovery period; Fig. S5). Leaf Chl content was estimated using a Chl concentration metre (MC‐100; Apogee, Maidstone, UK) by measuring three positions on the central leaflet for each plant. Plant height was measured using a ruler from the soil level to the apical meristem. Leaf length and width (widest part perpendicular to the length) were also measured with a ruler for the central leaflet only. Leaf thickness was measured with a digitronic calliper 110‐DBL (Moore & Wright). Total aboveground biomass was determined on a separate set of plants by drying leaves and stems at 60°C until constant weight.

Chl fluorescence (method described later) measurements were conducted every day of the heatwave. Three 0.38‐cm^2^ leaf discs were excised and quickly placed with the adaxial surface upwards on a single layer of wet filter paper on a thin plastic tray (< 0.5 mm) and immediately transported to the fluorescence imager.

A thermal imaging camera (CAT S60 smartphone; Caterpillar, Peterborough, UK) was used before sampling to assess *T*_leaf_. Each image was later analysed with the FLIR Thermal Studio software (Teledyne FLIR LLC, Newcastle‐under‐Lyme, UK), providing the mean temperature across an area of the leaf surface (Fig. S6).

For RNA‐seq analyses (method described later), four 0.55‐cm^2^ leaf discs were collected using a cork borer from one leaf per plant. Four independent biological replicates (plants) were used per treatment per time point (Days 1, 3 and 5 of heatwave), and samples were immediately snap‐frozen in liquid nitrogen and stored at −80°C.

For protein and Rubisco analyses, four 0.55‐cm^2^ leaf discs were excised as described previously and quickly placed with the adaxial surface upwards on a single layer of wet filter paper to maintain turgidity. The filter paper was contained in a thin plastic tray and positioned on the surface of the water in a circulating water bath in the artificial sunlight simulation rig (Taylor *et al*., 2022). Photosynthetic Photon Flux Density (PPFD) at leaf disc level was 850 μmol m^−2^ s^−1^. Water bath temperature was maintained at 26°C or 32°C (±0.1°C) for control or heat‐treated plants, respectively, to mimic leaf temperatures in the growth cabinet. After 1‐h incubation, leaf discs were quickly blotted on absorbent paper to remove excess moisture, then immediately frozen in liquid nitrogen and stored at −80°C.

For Rubisco reactivation in LEs, eight 0.55‐cm^2^ leaf discs were sampled on Day 5 of the heatwave. A different plant was used for each of the seven temperatures of the temperature response curve (20, 25, 32, 38, 40, 45°C). Samples were quickly frozen in liquid nitrogen, and then stored at −80°C until further analysis.

### Chlorophyll fluorescence

Samples were analysed using a Chl fluorescence imager (closed 800C FluorCam; Photon System Instruments, Drásov, Czech Republic). The imager contained a customised temperature‐controlled sample surface area, which comprised an aluminium plate with imbedded copper piping connected to a circulating temperature‐controlled water bath (Optima TX150; Grant Instruments, Royston, UK). The filter paper was in contact with sponges, which acted as water reservoirs to ensure the paper remained wet during analysis (Fig. S7a). The temperature of the sample surface area and leaf discs was recorded using type k thermocouples and a data logger (RDXL4SD; Omega, Manchester, UK). Additional thermocouple wires were placed in contact with the sample surface area and extra leaf discs to record temperature throughout the analysis (Fig. S7a). Samples were first dark‐adapted for 1 h at 26°C. After dark adaptation, the *F*_v_/*F*_m_ protocol in the software (FluorCam 7; Photon System Instruments) was used to make measurements of the maximum quantum efficiency of photosystem II (PSII) over a range of temperatures. The temperature of the leaf discs was used to assess when the measurement temperature had been reached and was stable. Once stable, the temperature was held for 3 min before the *F*_v_/*F*_m_ measurement was taken.

The data for each set of three leaf discs from the same plant were averaged, and plateauing *F*_v_/*F*_m_ points at higher temperatures were removed before the remaining data were used to calculate *T*_crit_ (Fig. S7b) with the segmented function from the segmented package (Muggeo, 2017) in R. This function uses a previously fitted linear model to identify a breakpoint and fits two linear models before and after the breakpoint. The breakpoint in the *F*_v_/*F*_m_ curve was taken as *T*_crit_, while the slopes of the two linear models were taken as m1 & m2 (Fig. S7b–d).

### Protein extraction, Rubisco activity and content

Proteins were extracted from leaf disc samples, and Rubisco activity assays were performed in a randomised order, as described previously (Taylor *et al*., 2022; Ashton *et al*., 2024). The activation state of Rubisco was calculated as the ratio of initial to total activity. In short, aliquots of the LE supernatant were added to an assay mix containing ^14^CO_2_ and RuBP to initiate assays that gave an estimate of Rubisco initial activity, whereas Rubisco total activity was measured after allowing a 3‐min incubation period of the LE in the assay mix without RuBP to allow full carbamylation of Rubisco.

Aliquots of LE supernatant were also used for determining Rubisco content via ^14^2‐carboxy‐d‐arabinitol 1,5‐bisphosphate binding, as previously described (Ruuska *et al*., 1998; Ashton *et al*., 2024) and total soluble protein (TSP) content using the Bradford method (Bradford, 1976).

### Gene expressions analysis

Frozen leaf disc samples were ground to a fine powder in liquid nitrogen using a pestle and mortar. Total RNA was extracted from 20 to 30 mg tissue using a NucleoSpin™ RNA Plant Kit (Macherey‐Nagel) including a DNase treatment, according to the manufacturer's instructions. RNA concentration and quality were determined by spectrophotometry using a microplate reader and LVis plate (SPECTROstar Nano; BMG Labtech) (Table S5). Further quality control (QC) analysis of RNA samples was performed by Novogene using a Qubit fluorometer (Invitrogen) and an Agilent 5400 fragment analyzer, followed by mRNA purification (using poly‐T oligo‐attached magnetic beads), library construction and 2 × 150‐bp paired‐end (PE150) sequencing using an Illumina NovaSeq 6000 (read depth of 30 million reads).

An initial filtering was applied to the raw data to remove reads containing adapters, containing *n* > 10% (*n* represents bases that cannot be determined) and low‐quality bases (Phred <5). Further read quality assessment and trimming were performed using the FastQc tool, v.0.12.1 (http://www.bioinformatics.babraham.ac.uk/projects/fastqc/) and cutadapt, v.4.2 (Martin, 2011). Low‐quality bases (Phred < 30) were trimmed from 3′ ends of each read, flanking N bases were removed, and trimmed reads shorter than 50 bp were discarded (for sequencing and alignment statistics, see Table S6).

Cowpea IT97K‐499‐35 reference transcriptome v.1.2 (Lonardi *et al*., 2019; Liang *et al*., 2024) was downloaded from Phytozome 13 (Goodstein *et al*., 2012). Salmon, v.1.10.1 (Patro *et al*., 2017) was first used to index the reference transcriptome and then quantify expression of clean reads at the transcript level. To identify changes in the cowpea Rca transcripts, transcripts per million (TPM) values were extracted from the Salmon Quant.sf output files for Rca, RbcS and HSP20 transcripts. Three transcripts are present for Rca1β (the first two result in the same protein, the third is missing some of the N domain), and TPM values for all three were combined. Three transcripts exist for Rca8α. Transcripts 2 and 3 are nonfunctional and have low expression so here we only present data for the first transcript. For Rca10, Transcripts 1, 3 and 4 are combined to give total expression for the alpha isoform, and Transcript 2 alone represents the beta isoform. For RbcS, there are two genes on Chromosomes 3 and 4; the former only produces negligible transcript levels of expression, while the latter produces high levels of one transcript and low levels of two other transcripts – the three transcripts encoded by the gene on Chromosome 4 produce an identical protein and were combined.

Global gene expression analysis was carried out in R v.4.4.1 (2024‐06‐14, Race for Your Life). Raw counts from Salmon were loaded into EdgeR 4.2.1 using tximport 1.32.0 and rtracklayer 1.64.0. Transcript data for each gene were combined at this stage so that all global analysis was performed at the gene level. Principal component analysis plot was made using logCPM and the PCAtools 2.16.0 to check that biological replicates group together and to look for outlying samples. Here, we used the counts per million (CPM) to remove the variability due to library depth. The EdgeR trimmed mean of M‐values method was used for normalisation of read counts. These data were filtered using a CPM threshold to remove genes with zero or near‐zero expression. A generalised linear model was then fitted to the normalised and filtered data for each gene. Empirical Bayes quasilikelihood *F*‐tests were used to test whether genes were significantly differentially expressed between heat‐treated and control plants on each day of the heatwave. To control for multiple testing, *P*‐values were calculated using the false discovery rate (FDR) of Benjamini–Hochberg. Differentially expressed genes were then identified using the threshold values of a FDR of < 0.05 and a log_2_ (|fold change|) of > 1.5.

Rca gene expression changes were validated via reverse transcription quantitative polymerase chain reaction (RT‐qPCR) (Fig. S8) to confirm transcript profiles of Rca isoforms. Sampling and detailed methodology is given in Tables S7 and S8.

### Rca quantification via western blotting

To quantify the abundance of different Rca protein isoforms, an aliquot of supernatant resulting from Rubisco analysis was mixed with SDS‐PAGE loading buffer and separated as previously described in Perdomo *et al*. (2018) on a 12% TGX Stain‐Free gel (Bio‐Rad). Gels were imaged to assess total protein before transfer to a nitrocellulose membrane using a dry blotting system (iBlot2; Thermo Fisher Scientific, Morecambe, UK). Membranes were stained with REVERT Total Protein Stain for normalisation (Li‐Cor Biosciences, Cambridge, UK) and imaged before application of antibodies. To compare the abundance of α and β isoforms of Rca, an antibody with broad specificity for both isoforms was used (Feller *et al*., 1998; a gift from Michael Salvucci), with detection via a secondary fluor‐tagged antibody (IRDye800CW; Li‐Cor Biosciences, RRID:AB_1660973). A dilution series of a pooled sample was run on every gel alongside the samples to verify antibody detection was within the linear range. To quantify Rca8α, a more specific antibody was used (Abcalis) in place of the broad specificity antibody. The anti‐Rca8α‐specific antibody was generated using phage display with the peptide selected targeted against KRGAFYGKAAQQINVP (amino acid residue 376–391), as described in Bloemers & Carmo‐Silva (2024). All blot images were obtained using an Odyssey FC (Li‐Cor Biosciences). The Empiria studio software (v.2.3.0.154; Li‐Cor Biosciences) was used for image data analysis and normalisation with the total protein stain, with values determined as signal intensity.

### Characterisation of Rca thermal optima in leaf extracts

Rubisco reactivation by Rca in LEs was determined as described in Carmo‐Silva & Salvucci (2011). Eight leaf discs per plant were sampled from the youngest fully expanded leaf on Day 5 of the heatwave. Separate samples were used for assaying each of the seven temperatures of the temperature–response curve as shown in Fig. 4, and eight plant sample replicates were used per temperature. Samples were stored at −80°C until further analysis. One replicate of samples from one cabinet at control and one cabinet at high temperature was used on each day of Rca assays, with samples allocated randomly to each assay temperature. Fig. S9 illustrates the method that uses LEs as the source of both Rubisco and Rca, incubates subaliquots with different assay mixes and then carries out a two‐stage assay very similar to the assay with the purified proteins. This enables the determination of the capacity of a population of Rca, drawn from plants grown in different conditions, to reactivate Rubisco. Model fitting and temperature parameter (*T*_max_, *T*_opt_ and *T*_0.5_) calculations were conducted as described previously for the *in vitro* Rca activity assays, and Rca activity was normalised on TSP. Aliquots of the desalted extract were used for TSP content determination using the Bradford method (Bradford, 1976).

### Data analyses

**Fig. 1:**
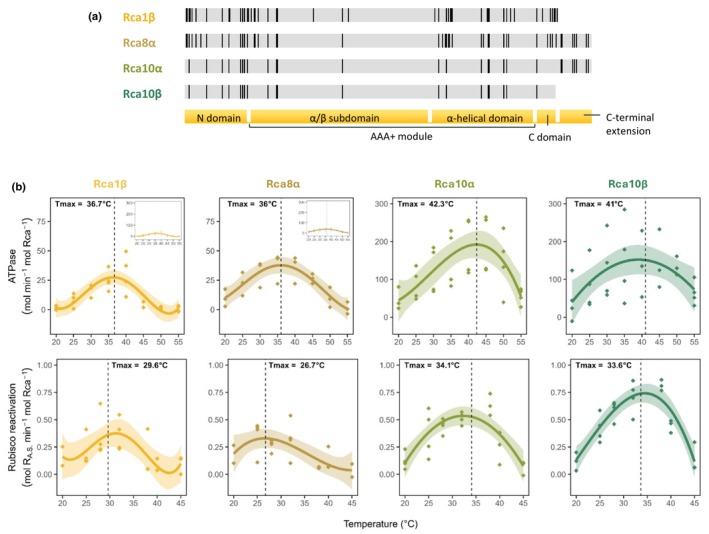
Temperature response of cowpea Rubisco activase (Rca) isoforms *in vitro*. (a) Amino acid residue alignment of the four cowpea (*Vigna unguiculata*) Rca isoforms encoded by genes located on Chromosomes 1, 8 and 10. Black vertical lines indicate differences in sequence among the genes. The following corresponding regions of the protein are shown: N domain, α : β subdomain, α‐helical domain, C‐domain and C‐terminal extension. (b) Temperature response of ATP hydrolysis and Rubisco reactivation by cowpea Rubisco activase isoforms *in vitro*. Rate of ATP hydrolysis and Rubisco reactivation by recombinantly produced purified cowpea Rca isoforms Rca1β, Rca8α, Rca10α and Rca10β. For Rca1β and Rca8α, subplots are included with the ATPase temperature response plotted with the y‐axis on the same scale as the isoforms Rca10α and Rca10β. Dotted vertical line denotes *T*_max_. Assays were performed at the indicated temperatures using 1 μM purified Rca and 5 mM ATP. Activation of pre‐inhibited Rubisco used 5 μM Rubisco active sites (R_A.S._) in the ER form (1 : 5 Rca : R_A.S_). Rubisco activation is presented relative to maximum Rubisco activity, determined as the Rubisco activity when Rca is allowed to reactivate inhibited Rubisco for 5 min. Values represent biological replicates (independent purifications; *n* = 3–4 biological replicates). Lines represent the best fit for each enzyme (selected by the Akaike information criterion), and shaded areas denote the 95% confidence interval for each fit (Supporting Information Table S3).

Geneious 2024.0.7 was used to generate protein alignments as depicted in Fig. 1(a). Data were processed using R 4.3.1 and RStudio 2024.09.0. Graphs were prepared using the ggplot 2 (Wickham, 2017), EnhancedVolcano (Blighe *et al*., 2018), VennDiagramm (Chen & Boutros, 2011), ggpubr (Kassambara, 2023) and patchwork (Pedersen, 2024) R packages. Outliers were detected before statistical analysis with the outliers package using Tukey's fences method, in which outliers are defined as extreme values that are 1.5 times the interquartile range (1.5 IQR) below the first quartile or 1.5 IQR above the third quartile. Box plots show medians and the first and third quartiles (25^th^ and 75^th^ percentiles), and whiskers extend from the hinge to the largest or smallest value. Symbols represent individual data points (technical or biological replicates defined for each plot either on methods or figure legend). Bar plots show means and error bars represent standard error. For statistical analysis, normality of data was assessed using the Shapiro–Wilk test and equality of variances using the Levene test. For measurements on multiple days, two‐way ANOVA tests were applied to determine whether there were significant day, treatment or interactive effects, followed by *post hoc* Tukey's tests for multiple comparisons with Bonferroni correction. Compact letter display was generated after the *post hoc* pairwise comparisons using packages multicomp (Bretz *et al*., 2016) and multicompview (Graves *et al*., 2019). In cases where there was a slight violation of normality or homogeneity of variance when data between different days were compared, two‐way ANOVA was still chosen as the most robust method. If normality and variance were severely violated, data were log‐transformed, and two‐way ANOVA was applied to the transformed data provided assumptions were not severely violated in the transformed data. For the Rca isoform α : β ratio, data were log‐transformed and analysis used the geometric mean. For measurements taken at a single time point (growth parameters), *t*‐test was used to determine statistical significance between treatments. Statistical analysis results and full datasets are available via Zenodo repository.

## Results

### Cowpea contains four Rubisco activase isoforms

The cowpea genome contains three genes that encode Rca. The gene on Chromosome 1 (*Vigun01g219300*) encodes an Rca β, the gene on Chromosome 8 (*Vigun08g150700*) encodes an Rca α, and the gene on Chromosome 10 (*Vigun10g051600*) encodes both Rca α and β isoforms via alternative splicing (Fig. 1a). The gene on Chromosome 8 also encodes a truncated version of Rca; however, this would not produce a canonical, fully functional Rca as it has a premature stop codon before the α‐helical domain, which is necessary for the recognition of Rubisco by Rca (Portis *et al*., 2008). Rca isoforms in cowpea exhibit minor sequence differences, primarily in the N‐terminal region (the flexible domain critical for Rubisco binding) (Stotz *et al*., 2011; Shivhare *et al*., 2019) and the α‐helical domain (Fig. S1).

### Cowpea Rca isoforms differ in thermal optima of activity *in vitro*

Previous studies have shown the potential for intraspecies variation of Rca thermal optima (Degen *et al*., 2020). With four distinct isoforms in cowpea and its known ability to grow in warm (20–37°C) temperatures (Barros *et al*., 2021), recombinant versions of each isoform were produced for *in vitro* characterisation of cowpea Rca thermal optima (Fig. 1b). Both enzyme activities were assessed, that is the ability to reactivate inhibited cowpea Rubisco and the ATPase activity that drives reactivation (Robinson & Portis, 1989). Rubisco reactivation by Rca was calculated after accounting for the spontaneous reactivation of Rubisco, expressed as a percentage of maximum Rubisco activity. The spontaneous reactivation of Rubisco, although insignificant at lower temperatures, increases with temperature (Fig. S2) as the Rubisco catalytic site becomes more flexible. For all the Rca isoforms, the temperature corresponding to the peak of activity (*T*_max_), the range of temperatures at which activity was maintained above 70% of the maximum value (*T*_opt_) and the temperature at which activity drops below 50% of the maximum (*T*_0.5_) were consistently higher for ATPase activity than for Rubisco reactivation activity (Table 1). A higher sensitivity of Rubisco reactivation compared with ATP hydrolysis has been previously reported and may be due to the interaction with Rubisco being the key step impacted by elevated temperatures in Rca (Degen *et al*., 2020). It has also been shown that while ATP hydrolysis can be catalysed by Rca oligomers consisting of three subunits, Rubisco reactivation requires oligomers containing more than three subunits (Keown *et al*., 2013; Hazra *et al*., 2015).

**Table 1:** Temperature response of *in vitro* Rubisco activase (Rca) activity calculated parameters.

Rca isoform	ATPase_max_ (mol min^−1^ mol Rca^−1^)	*T*_max_ (°C)	*T*_opt_ (°C)	*T*_0.5_ (°C)
1β	27.9 ± 5.5	36.7 ± 0.5	29.8–43.3	45.6 ± 1.1
8α	35.4 ± 5.5	36.0 ± 0.8	25.9–46.1	49.1 ± 0.5
10α	193.8 ± 39.3	42.3 ± 1.0	31.7–50.8	53.0 ± 0.6
10β	185.5 ± 58.2	41.0 ± 1.2	29.8–52.1	53.5 ± 0.3

Rca isoform	Rubisco reactivation_max_ (mol R_A.S._ min^−1^ mol Rca^−1^)	*T*_max_ (°C)	*T*_opt_ (°C)	*T*_0.5_ (°C)
1β	0.38 ± 0.12	29.6 ± 0.8	25.6–34.6	36.7 ± 0.9
8α	0.39 ± 0.07	26.7 ± 0.9	23.4–31.5	33.7 ± 0.0
10α	0.54 ± 0.02	34.1 ± 0.8	26.4–40.6	42.4 ± 0.2
10β	0.82 ± 0.07	33.6 ± 0.2	26.6–40.1	42.0 ± 1.1

Maximum rate of ATP hydrolysis and Rubisco activation and corresponding temperature (*T*_max_), optimum temperature range (*T*_opt_, above 70% activity) and temperature above the optimum at which 50% of the maximum activity remains (*T*_0.5_) of the *in vitro* temperature response of the four cowpea (*Vigna unguiculata*) Rca isoforms. Values were estimated from best‐fit models that describe the *in vitro* temperature response of each Rca isoform selected by the Akaike information criterion (Supporting Information Table S3). The best‐fit model was applied to each of three biological replicates, with complete temperature response curves shown in Fig. 1(b). The first biological replicate was excluded from the fitting of the individual replicates because the temperature response of Rca activity was incomplete (determined only up to 32°C); this replicate is included in the model fitting to the complete temperature response data set (Table S4). Values shown here are the mean ± SE of the mean of values determined for individual replicates of temperature response curves (*n* = 3).

The two Rca isoforms encoded by *Rca10* were faster at hydrolysing ATP and reactivating Rubisco, had higher thermal maxima (*T*_max_), broader thermal optima (*T*_opt_) and showed thermal sensitivity (*T*_0.5_) at warmer temperatures compared with the other two isoforms (Fig. 1b; Table 1). *T*_max_, *T*_opt_ and *T*_0.5_ calculated for both ATP hydrolysis and Rubisco reactivation were generally similar for Rca1β and Rca 8α (Table 1), but Rca8α exhibited the lowest *T*_0.5_ for reactivation activity (33.7 ± 0°C). Up to 33.5°C, all Rca isoforms maintained at least 70% of maximum Rubisco reactivation activity. Rca10α exhibited the highest temperature corresponding to the peak of activity *in vitro* for both ATP hydrolysis (*T*_max_ = 42.3 ± 1°C) and Rubisco reactivation (*T*_max_ = 34.1 ± 0.8°C). *In vitro T*_max_ values for Rca10α and Rca10β were at least 3°C higher for both ATP hydrolysis and Rubisco reactivation relative to Rca1β and Rca8α (Table 1). Combined, the results show that the cowpea Rca10α and Rca10β isoforms have faster rates, higher thermal maxima, broader thermal optima and are less sensitive to warmer temperatures than Rca1β and Rca8α.

### A 5‐d heatwave of +10°C causes mild heat stress in cowpea

**Fig. 2:**
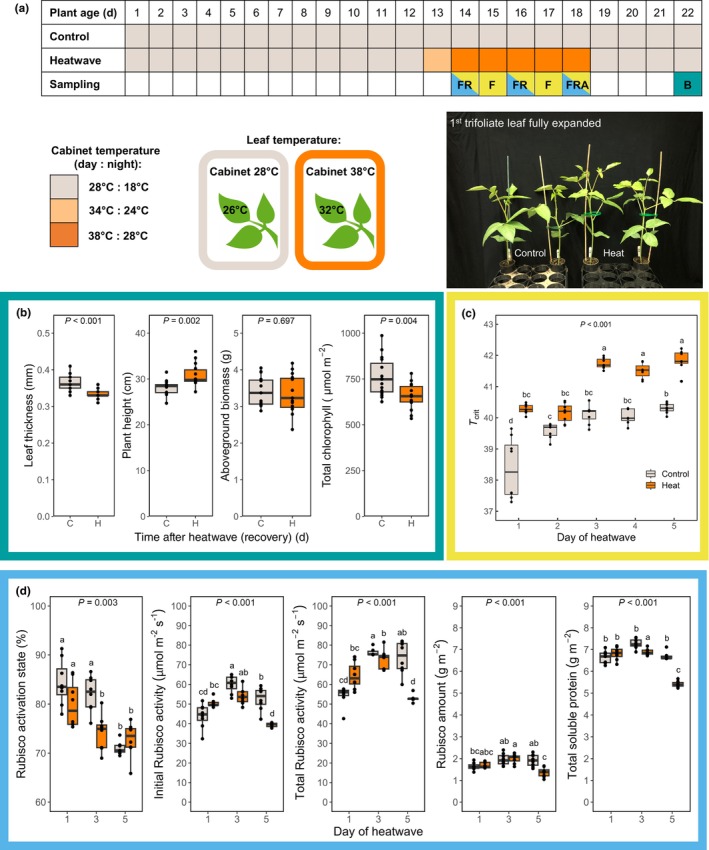
Cowpea response to a heatwave in controlled environment. (a) Experimental design, heatwave and sampling times. Cowpea (*Vigna unguiculata*) plants were grown at 28°C : 18°C, day : night for 13 d, then exposed to an intermediate heat treatment for a day at 34°C : 24°C, day : night before being exposed to a 38°C : 28°C, day : night heatwave for 5 d. Sampling: (F) photosystem II (PS II) maximum efficiency (*F*_v_/*F*_m_) was measured daily during the heatwave (yellow); (R) leaf discs for RNA‐seq analysis, total soluble protein (TSP), Rubisco activity and amount were sampled on Days 1, 3 and 5 of the heatwave (blue); samples for Rca temperature response in leaf extracts (LEs) were taken on Day 5 of heatwave; growth parameters and biomass were measured on Day 22 of plant growth, 4 d after the end of the heatwave (recovery) (green). Cabinet and leaf temperature were monitored daily. (b) Leaf and whole plant growth parameters were measured 4 d after the end of the heatwave (*n* = 15). Relative Chl content was determined using a concentration metre based on leaf reflectance. (c) *T*_crit_ was calculated for each day of the heatwave as the breaking point where the two *F*_v_/*F*_m_ slopes meet (Supporting Information Fig. S7). (d) Rubisco activation state (*n* = 7–8), initial (*n* = 7–8) and total activity per leaf area (*n* = 6–8), Rubisco amount (*n* = 7–8) and TSP (*n* = 6–8) were measured in LEs on Days 1, 3 and 5 of the heatwave. Box plots show medians and the first and third quartiles (25^th^ and 75^th^ percentiles), and whiskers extend from the hinge to the largest or smallest value. Symbols represent individual data points (biological replicates). *P*‐values were calculated using two‐way ANOVA followed by Tukey's *post hoc* tests.

To investigate heat‐induced changes in cowpea Rca *in planta*, young plants were exposed to a 5‐d heatwave consisting of an increase of 10°C during both day and night. Plants were cultivated for 2 wk at 28°C : 18°C, day : night, then in half of the controlled environment cabinets the temperature was increased to 34°C : 24°C, day : night for 1 d and subsequently to 38°C : 28°C, day : night for 5 d (Figs 2a, S3, S4). The intermediate day aimed to resemble a gradual increase in temperature in field conditions. Plants were well‐watered throughout the heatwave as the focus was on the impact of heat on Rca and we wanted to avoid confounding effects of water deficit on CO_2_ diffusion and assimilation. Cabinet and leaf temperatures were monitored throughout the experiment to ensure the plants were under heat stress conditions, with heat‐treated plants showing a 6°C increase in leaf temperature to 32°C (Figs 2a, S6). Thus, plants experiencing the heatwave had leaf temperatures within the *T*_opt_ of Rca activity.

Plant growth traits were determined 4 d after the heatwave (Day 22 of growth), after plants were allowed to recover, and showed that the 5‐d heatwave of +10°C had only a mild impact on cowpea (Fig. 2b). Exposure to elevated temperature resulted in decreased leaf thickness and increased plant height; however, no effect on aboveground dry biomass was observed. Total Chl also decreased in leaves that had been exposed to heat when determined 4 d after the heatwave (Fig. 2b). Measurement of leaf and plant traits during the heatwave suggested an initial increase in the rate of cowpea leaf and plant growth (Day 2; Fig. S5), but only minimal differences between control and heat at the end of and after the heatwave.

As a proxy for photosynthetic activity, PSII maximum efficiency (*F*_v_/*F*_m_) was monitored throughout the heatwave (Fig. S7). Changes in *F*_v_/*F*_m_ indicate modifications in the functionality of PSII reaction centres, with a decrease indicating photooxidative damage and reduced photochemical quantum yield (Murchie & Lawson, 2013). *F*_v_/*F*_m_ data collected from plants throughout the heatwave enabled the determination of *T*_crit_ (Fig. 2c), which corresponds to the temperature at which Chl*a* fluorescence rises rapidly in response to temperature due to thermal damage in PSII and is used as an indicator of photosynthetic thermal stability (Perez & Feeley, 2020; Posch *et al*., 2022). *T*_crit_ slightly increased in control plants from Day 1 to Day 3 of the experiment as the leaf developed (Fig. 2c). In heat‐treated cowpea, *F*_v_/*F*_m_ did not decline compared with control plants (Fig. S7e), indicating that no irreversible damage was caused to PSII reaction centres due to heat stress. In fact, heat‐treated plants showed a progressive increase in the temperature at which *F*_v_/*F*_m_ decreased, resulting in a significantly higher *T*_crit_ after 3 d, denoting increased PSII thermal stability (*P* < 0.001; Fig. 2c). These results indicate a dynamic response that allows for PSII efficiency to adapt to warmer conditions.

### Heat stress induces a decline in Rubisco activation state

In contrast to *T*_crit_, the activation state of Rubisco decreased during the heat treatment (Fig. 2d). Importantly, a decline in Rubisco activation was observed from the beginning to the end of the experiment as leaves aged. This decline was observed earlier in heat‐treated plants (Day 3) compared with that in control plants (Day 5), indicating that heat stress accelerated this decline in Rubisco function. Rubisco activation state, defined as the ratio of initial to total Rubisco activity, reflects the enzyme's operational efficiency under different conditions. Initial activity represents the enzyme's immediate functionality upon extraction, while total activity reflects its potential after full carbamylation (Ashton *et al*., 2024). Although Rubisco activation state was similar on Day 5, the initial and total activity was significantly lower in heat‐treated than in control plants (*P* < 0.001; Fig. 2d). This decrease in Rubisco activity on Day 5 of the heatwave was associated with decreased Rubisco amount and TSP (Fig. 2d), as no significant differences were detected in leaf thickness during the heatwave nor in Rubisco specific activity, expressed per quantity of Rubisco protein (Figs S5, S10).

### Heat stress induces the expression of thermotolerant Rca isoforms

**Fig. 3:**
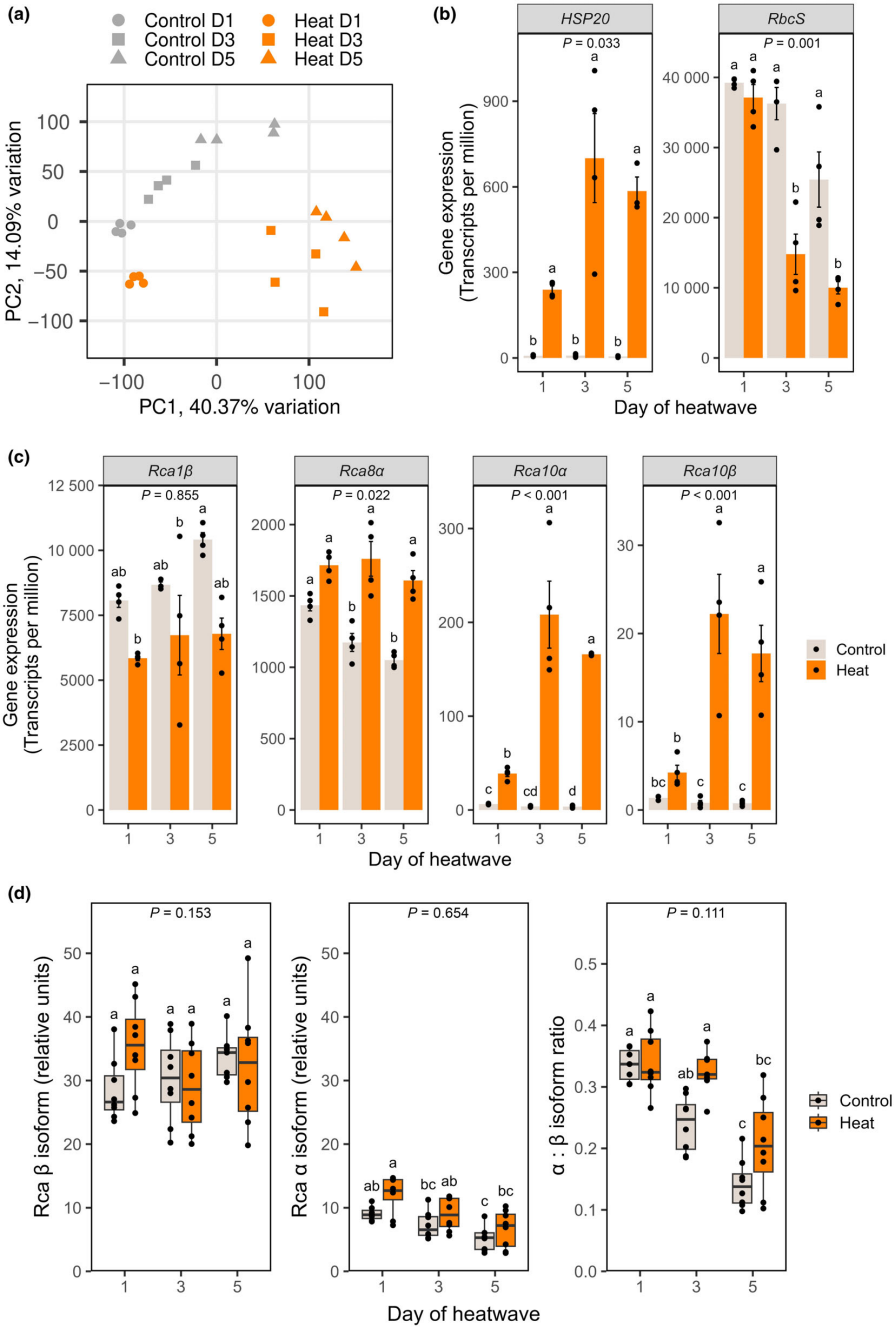
Differential gene expression and Rca protein abundance in cowpea during a heatwave. (a) Principal components analysis (PCA) plot of gene expression in leaves of heat‐treated and control cowpea (*Vigna unguiculata*) plants on Days 1, 3 and 5 of the heatwave. (b) Expression levels of the heat shock protein 20 (HSP20) and the small Rubisco subunits (RbcS) were used as reference. Bar plots represent mean, and error bars represent SE. (c) Expression levels of the four cowpea Rubisco activase (Rca) isoforms in control and heat‐treated plant on Days 1, 3 and 5 of the heatwave (*n* = 4). (d) Protein abundance of Rca isoforms in leaves of control and heat‐treated plants on Days 1, 3 and 5 of the heatwave (relative units). Rca α and β isoforms were detected using an anti‐Rca antibody and normalised to total protein. The size difference between the α (45 kDa) and β (41 kDa) isoforms enabled the identification as separate bands, and the estimation of the α : β ratio. Box plots show medians and the first and third quartiles (25^th^ and 75^th^ percentiles), and whiskers extend from the hinge to the largest or smallest value. Bar plots show mean, and error bars represent SE. Symbols represent individual data points (biological replicates). *P*‐values were calculated using two‐way ANOVA followed by Tukey's *post hoc* tests. For the α : β ratio, ANOVA was performed after log transformation and using the geometric mean. Samples were run in separate gels and normalised to total protein via reversible staining of the membrane.

Potential heat‐induced changes in gene expression of *Rca* isoforms were assessed through RNA sequencing (RNA‐seq) analysis on Days 1, 3 and 5 of the heatwave. A principal component analysis (PCA) distinguished gene expression changes caused by heat stress and those resulting from leaf aging (Figs 3a, S11). Heat‐induced differential gene expression occurred from the first day of the heatwave, with both up‐ and downregulation of genes evident in heat‐treated plants compared with that in control conditions throughout the duration of the heatwave (Fig. S11). Heat stress was confirmed by the upregulation of the heat shock protein *HSP20* (Song *et al*., 2017) from Day 1 of the heatwave (Fig. 3b). The heatwave also decreased expression of the Rubisco small subunit (*RbcS*), which is likely associated with the lower Rubisco amounts (Fig. 3c).

Under control temperature conditions, *Rca1β* was found to be the most abundant Rca transcript, followed by *Rca8α*, while *Rca10α* and *Rca10β* were expressed at much lower levels (Fig. 3c). Of the hundreds of up‐ and downregulated genes (Fig. S11), volcano plots showed that *HSP20* and *Rca10* were both upregulated on Days 1, 3 and 5 of the heatwave (Fig. S12). Heat‐treated plants showed a (nonsignificant) tendency for lower *Rca1β* expression, whereas *Rca8α*, *Rca10α* and *Rca10β* were all upregulated during the heatwave (Fig. 3c). It is noteworthy that despite the upregulation of both *Rca10* transcripts, their expression levels remained much lower than *Rca1β* and *Rca8α* (Fig. 3c). Changes in *HSP20* and the four *Rca* transcript levels were also confirmed via RT‐qPCR (Fig. S8). Moreover, promoter analysis revealed multiple stress‐responsive elements in *Rca1β and Rca8α*, while *Rca10* contains two heat stress response elements and has fewer other stress‐responsive elements (Fig. S13).

To investigate whether changes in gene expression are reflected at the protein level, the abundance of leaf Rca protein was quantified via immunoblotting (Fig. 3d). A pan‐Rca antibody that reacts with all isoforms of Rca and enables quantification of Rca α and Rca β isoforms, as these run separately in gel electrophoresis due to the difference in molecular weight, was used to identify changes in abundance between α and β isoforms (Perdomo *et al*., 2018). While differential gene expression was observed, changes in Rca protein were less pronounced. As *Rca10β* transcript levels are almost negligible compared with *Rca1β*, it is reasonable to infer that the β isoform protein is predominantly attributable to *Rca1β* expression. The expression of *Rca8α* is also likely to produce most of the α isoform protein. Rca β remained unchanged during the heatwave, while the α isoform decreased from Day 1 to Day 5 but was not significantly different between control and heat‐treated plants (Fig. 3d). Protein abundance of Rca8α was also determined using a specific antibody that only reacts with this isoform (Bloemers & Carmo‐Silva, 2024). Rca8α showed a near identical pattern to the pan‐Rca antibody for Rca α with a nonsignificant tendency for increased abundance in heat‐treated plants compared with the control (Fig. S14a). The Rca isoform α : β ratio was lower on Day 5 than on Day 1 for both control and heat‐treated plants, and although no significant difference was found between treatments, a trend can be seen for heat‐treated plants to maintain a higher α : β ratio on Days 3 and 5 (Fig. 3d). Due to their very low abundance, Rca10α and Rca10β protein isoforms were undetected in LEs with Rca10‐specific antibodies (Fig. S14b).

### Heat‐treated plants maintain Rca rate and thermal optimum

**Fig. 4:**
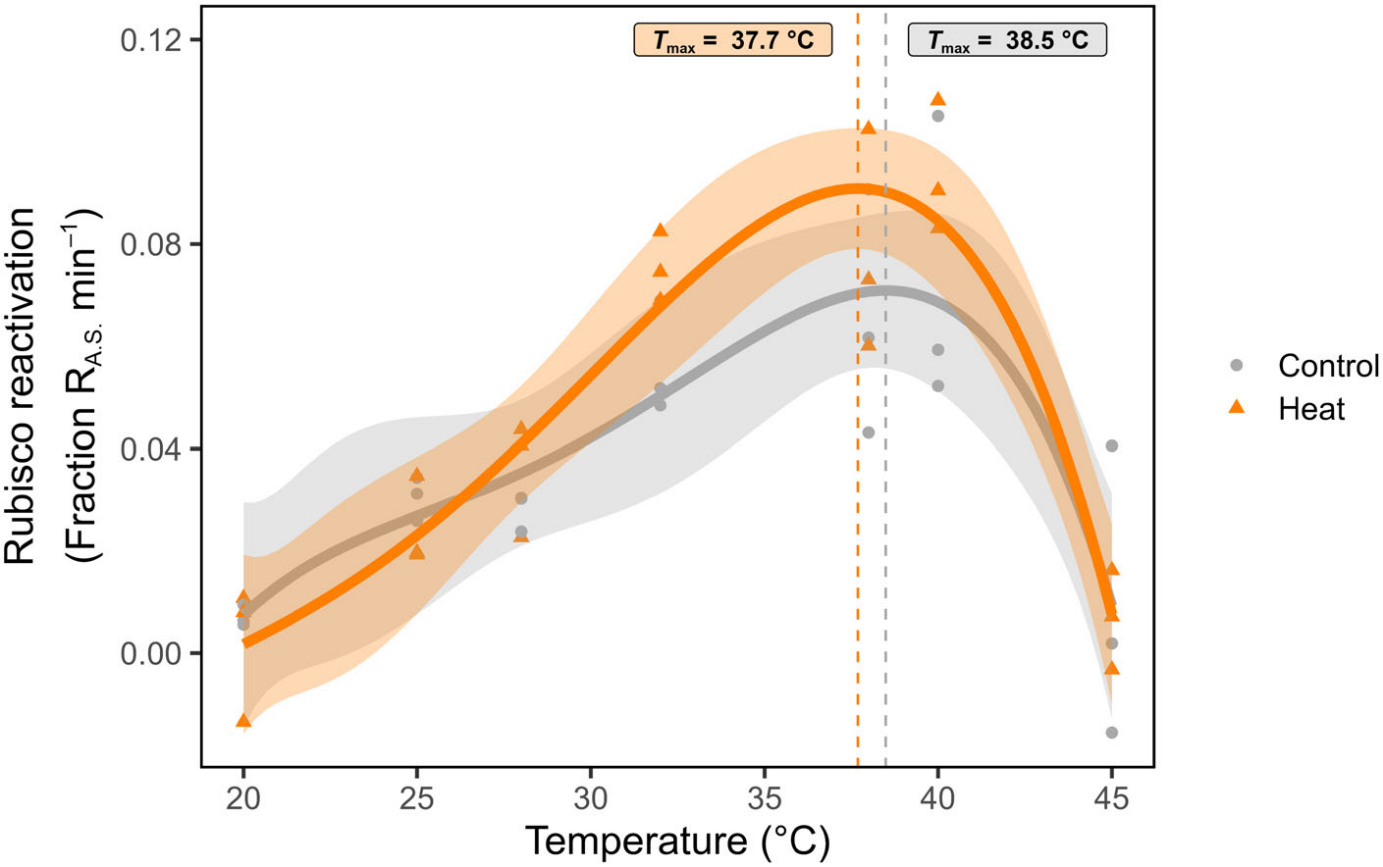
Temperature response of Rca activity in leaf extracts (LEs) of control and heat‐treated cowpea plants. Leaf discs of control and heat‐treated cowpea (*Vigna unguiculata*) plants were sampled on Day 5 of the heatwave. The Rubisco and Rca contained in the leaves of each plant were extracted, Rubisco was inhibited by binding of RuBP to decarbamylated catalytic sites in the absence of CO_2_ and Mg^2+^, then reactivation by Rca was measured at varying temperatures by comparing an assay in presence of ATP and ATP‐regenerating system with the negative control assay in the absence of ATP. The rate of Rubisco reactivation was calculated from measurements of Rubisco activity at 30 and 90 s after the start of the assay and expressed relative to maximum Rubisco activity in the same LEs (Supporting Information Fig. S9). Symbols represent biological replicates (LEs from independent plant samples; *n* = 6–8). Lines represent the best fit for each group of plants (selected by the Akaike information criterion), and coloured areas denote the 95% confidence interval for each fit (Table S9). Dashed lines correspond to the temperature in which Rca activity reaches maximum (*T*_max_).

To test the hypothesis that heat‐induced changes in the relative abundance of Rca isoforms are reflected in activity, we characterised the temperature response of Rubisco reactivation by Rca in protein extracts from leaves sampled on Day 5 of the heatwave (Fig. 4). LEs were desalted to produce the free Rubisco apoenzyme (uncarbamylated form), which was subsequently inhibited through incubation with RuBP (Carmo‐Silva & Salvucci, 2011). The inhibited Rubisco in the LEs was then used to determine the rate of reactivation by the Rca holoenzyme via removal of RuBP from uncarbamylated Rubisco catalytic sites, at a range of temperatures (Fig. S9). Concurrent measurement of spontaneous reactivation of Rubisco in the absence of ATP, which is required for Rca activity, enabled calculation of the spontaneous (Fig. S15) and Rca‐mediated rate of Rubisco reactivation (Fig. 4).

Rubisco reactivation by Rca tended to be faster in heat‐treated than in control plants; however, this increase was not statistically significant (*P = 0.339*, Fig. 4). Heat‐treated plants exhibited similar thermal optima of Rubisco reactivation by Rca to control plants at *c*. 38°C (Fig. 4). *T*_opt_ and *T*_0.5_ were also similar between control and heat‐treated plants (Table 2), indicating that the large fold‐change in *Rca10* transcript abundance (Fig. 3c) and the small change in heat‐induced protein Rca isoform ratio (Fig. 3d) did not alter the temperature profile of Rubisco reactivation by Rca in the leaves of heat‐treated plants (Fig. 4). Interestingly, the *T*_max_, *T*_opt_, and, to a lesser extent, *T*_0.5_ of Rubisco reactivation by Rca were higher in LEs (Table 2) than in the respective values measured for each individual Rca isoform purified after recombinant expression (Table 1).

**Table 2:** Temperature response of Rubisco activase (Rca) activity in leaf extracts calculated parameters.

Treatment	Rubisco reactivation_max_ (fraction R_A.S._ min^−1^)	*T*_max_ (°C)	*T*_opt_ (°C)	*T*_0.5_ (°C)
Control	0.07	38.5	31.9–42.6	43.4
Heat	0.09	37.7	31.4–42.2	43.3

Maximum rate of Rubisco activation and corresponding temperature (*T*_max_), optimum temperature range (*T*_opt_, above 70% activity) and temperature above the optimum at which 50% of the maximum activity remains (*T*_0.5_) of the temperature response of cowpea (*Vigna unguiculata*) Rca in leaf extracts. Values were estimated from best‐fit models selected by the Akaike information criterion that describe the temperature response of the pool of Rca isoforms extracted from leaves of control and heat‐treated cowpea on Day 5 of the heatwave (Supporting Information Table S9). The best‐fit model for each treatment was applied to combined replicates shown in Fig. 4 (*n* = 6–8).

## Discussion

### Cowpea Rca isoforms differ in thermal optima

We investigated the temperature response of Rubisco activase in cowpea, a vital crop for sub‐Saharan Africa that is adapted to warm temperatures, yet susceptible to the increasing pressures of global warming. Several studies demonstrated that Rca thermolability limits photosynthesis at elevated temperatures (Feller *et al*., 1998; Crafts‐Brandner & Law, 2000; Salvucci *et al*., 2006; Perdomo *et al*., 2017), making it a target for enhancing crop yield under elevated temperatures. In this study, we identified and characterised four Rca isoforms in cowpea: Rca1β, Rca8α, Rca10α and Rca10β. The relatively minor amino acid differences between the four sequences may contribute to functional diversity of Rca in cowpea, potentially allowing for fine‐tuning regulation of Rca in response to the environment. *In vitro* analysis across a range of temperatures revealed a higher thermal optimum for Rca10α and Rca10β for both ATPase and Rubisco reactivation activities (Fig. 1b). The most abundant Rca1β and Rca8α isoforms in cowpea leaves (Fig. 3) both exhibited lower thermal optima and thermal maxima, as well as slower rates of catalysis for both activities compared with the isoforms encoded by *Rca10* (Table 1).

Differences in thermal optima among isoforms have been reported for other species. In rice, the α isoform is more thermotolerant, while in wheat, one of the two β isoforms exhibits a higher thermal optima (Scafaro *et al*., 2018; Shivhare *et al*., 2019; Degen *et al*., 2020). For spinach, the β isoform is more sensitive to heat stress, with Rca β homohexamers displaying a lower thermal midpoint than Rca α homohexamers or heterohexamers comprising both α and β isoforms (Keown & Pearce, 2014). Notably, for all Rca isoforms, the *T*_max_ for ATPase activity is higher than for Rubisco reactivation, potentially due to differential oligomeric requirements for each activity. The proposed mechanism for Rca‐mediated Rubisco reactivation involves threading Rubisco through the AAA+ pore, akin to the method employed by AAA+ ATPases that adjust the conformation of misfolded proteins (Bhat *et al*., 2017; Houry *et al*., 2017; Waheeda *et al*., 2023). This mechanism requires hexameric Rca for Rubisco reactivation, while ATP hydrolysis can occur with three Rca subunits forming an oligomer (Keown *et al*., 2013; Hazra *et al*., 2015).

The maximum rates of both ATP hydrolysis and Rubisco reactivation were higher for the Rca10 isoforms than for Rca1β and Rca8α (Table 1). Similar differences among Rca isoforms were observed in rice and wheat but only for ATP hydrolysis, with the more thermostable Rca isoforms exhibiting lower Rubisco reactivation maxima than the less thermostable isoforms (Scafaro *et al*., 2016, 2019a; Shivhare & Mueller‐Cajar, 2017; Degen *et al*., 2020). In cowpea, we show that the increased thermostability of Rca10 isoforms occurs without a penalty in Rubisco reactivation rate. The broader *T*_opt_ of Rca10α and Rca10β spans and extends above the *T*_opt_ of Rca1β and Rca8α (Table 1). Thus, Rca10α and Rca10β appear to be ideal candidates to maintain CO_2_ assimilation by Rubisco both at optimal and supra‐optimal temperatures. Curiously, the promoter regions of both Rca1β and Rca8α contain multiple stress‐responsive elements while Rca10α and Rca10β have only two heat response elements (Fig. S13), suggesting an engineering approach may be required to alter the Rca isoforms predominantly involved in maintaining Rubisco activity *in planta*.

### Cowpea responds dynamically to a 5‐d 38°C : 28°C heatwave

A 5‐d heatwave at the vegetative growth stage (10°C increase to 38°C : 28°C, day : night) is considered moderate heat stress for most cowpea cultivars (Lonardi *et al*., 2019; Barros *et al*., 2021). Plants were well‐watered to avoid confounding effects of water deficit limiting the diffusion of CO_2_ to Rubisco sites (Carmo‐Silva *et al*., 2012). The leaf temperature only increased to 32°C (Fig. 2a), plant growth parameters were only mildly impacted, and the aboveground dry biomass remained unaffected (Fig. 2b,c). Notably, it has been shown that a heatwave during the vegetative stage can have a delayed impact on cowpea yield (Mohammed *et al*., 2024), which would not necessarily be expressed as reduced biomass at the end of the heatwave. *T*_crit_, an indicator of thermal lability of PSII, was higher on Day 5 of the 38°C : 28°C heatwave (Fig. 2c) suggesting some capacity for PSII thermal acclimation (Perez & Feeley, 2020). In wheat, increased *T*_crit_ after exposure to elevated temperature in field and controlled environment experiments correlated with a dynamic response of PSII to heat stress (Posch *et al*., 2022). On Day 5 of the 38°C : 28°C heatwave, cowpea plants had decreased Rubisco activities and amount, lower leaf thickness, total Chl and TSP (Fig. 2b). Similar results were observed in heat‐treated maize (Qu *et al*., 2023), which could have a delayed impact on biomass production.

### Thermotolerant Rca isoforms increase but remain low under heat

At optimal temperatures, *Rca1β* was the isoform most abundantly expressed, with *Rca8α* following, while *Rca10α* and *Rca10β* were only marginally expressed (Fig. 3c). Under heat stress, *Rca8α*, *Rca10α* and *Rca10β* expression was upregulated; however, *Rca10* transcript levels remained very low compared with *Rca1β* and *Rca8α*. The corresponding protein abundance was only marginally higher for Rca8α and remained undetectable for Rca10α and Rca10β (Figs S14, 3d). The Rca α : β ratio decreased as the leaves aged, and heat‐treated plants showed a trend to maintain a higher α : β ratio than control plants on Days 3 and 5, although this difference was not statistically significant (Fig. 3d). Variation in Rca α : β ratio under optimal and supra‐optimal temperatures may reflect differences in thermostability and regulatory mechanisms (Zhang & Portis, 1999; Wang *et al*., 2010; Degen *et al*., 2021). While Rca α is subject to redox regulation via thioredoxin‐f (Zhang & Portis, 1999; Shivhare *et al*., 2019; Kim *et al*., 2020) and tends to be more sensitive to inhibition by ADP (Zhang & Portis, 1999; Scafaro *et al*., 2019b; Amaral *et al*., 2024a), variation also exists in the properties of Rca β isoforms (Perdomo *et al*., 2019; Scafaro *et al*., 2019a).

Changes in relative abundance of Rca isoforms under heat stress are species‐specific (Yamori & von Caemmerer, 2009). In *Arabidopsis*, the two isoforms are comparable in thermotolerance and both increase with heat maintaining a 1 : 1 ratio (Ristic *et al*., 2009). Conversely, in rice and wheat, Rca isoforms differ in temperature response and heat induces upregulation of the thermotolerant variants (Scafaro *et al*., 2018, 2019a; Degen *et al*., 2021). While the same was observed for cowpea in the present study, the protein abundance of the thermostable Rca isoforms remained below detection levels in plants exposed to the 5‐d 38°C : 28°C heatwave. Overexpression of thermostable Rca via genetic engineering led to enhanced photosynthesis and yield (Kurek *et al*., 2007). Increasing Rca thermostability, and to a lesser extent upregulation of its quantity, has been shown to ameliorate the reduction in Rubisco activation state caused by elevated temperatures (Kurek *et al*., 2007; Scafaro *et al*., 2018, 2019a; Kim *et al*., 2020). The results presented here suggest that increasing Rca10α and Rca10β abundance in cowpea, while possible in longer or hotter heat conditions, would likely require an engineering approach.

### Rubisco reactivation by Rca in cowpea remains unaltered under heat

The temperature response of Rubisco reactivation in LEs of plants exposed to the 5‐d 38°C : 28°C heatwave showed a similar pattern and comparable thermal optima to control plants (Fig. 4). Scafaro *et al*. (2019a) reported increases in the Rubisco reactivation rate and *T*_0.5_ of Rca in LEs from heat‐treated wheat. In cowpea, the minor increase in rate was statistically insignificant, and *T*_opt_ and *T*_0.5_ were comparable between control and heat‐treated plants (Table 2). These results indicate that the heatwave in the absence of water deficit caused only mild heat stress, allowing plants to respond by adjusting isoform ratios in the leaf while preserving the Rca rate and temperature response.

Interestingly, the *T*_opt_ upper bound determined in LEs was 42°C for control and heat‐treated plants, much higher than the *T*_leaf_ in heat‐treated plants (Table 2; Fig. 2a). The Rca *T*_max_ in LEs (Table 2) was also higher than the Rubisco reactivation rate of each isoform measured *in vitro* (Table 1). Similar results were observed in rice (Scafaro *et al*., 2016). The higher Rca *T*_max_ in LEs may be attributed to several possible factors: differences in structural conformation of recombinant Rca, post‐translational modifications that regulate Rca activity in the leaf and are absent in recombinant proteins, heterohexamers comprising different isoforms *in planta* exhibiting higher thermal optima, or interaction of Rca with other components in the chloroplast. Chen *et al*. (2010) reported dynamic and reversible binding of rice Rca to the thylakoid membrane while it is active in remodelling the conformation of inhibitor‐bound Rubisco. Binding of Rca to the thylakoid membrane has also been reported during heat stress although its binding to the chloroplast ribosomes was proposed to have a protective role (Rokka *et al*., 2001). Moreover, heat stress has been shown to induce association of Rca with β‐subunit of chaperonin‐60 (cpn60β), a potential heat shock molecular chaperone (Salvucci, 2008). Thus, while *in vitro* analyses are useful to compare the properties of the individual isoforms, *in vivo* analyses as shown here for cowpea enable assessment of the overall Rca response within the chloroplast stroma.

### Conclusion

Cowpea is a vital source of protein for sub‐Saharan Africa, and despite being warm‐adapted, it is threatened by future heatwaves, which are likely to increase in intensity, duration and frequency. Here, we identified two isoforms of Rca that have higher thermal maxima, broader thermal optima and faster rates of ATP hydrolysis and Rubisco reactivation than the predominant Rca isoforms present in cowpea leaves. We showed that a 5‐d 38°C : 28°C heatwave in the absence of water deficit caused leaf temperature to increase to 32°C. Since this temperature is below the temperature at which Rca activity decreased below 70% of maximum for each Rca isoform, the impact of such a heatwave on Rubisco function and biomass production was only mild. However, cowpea plants growing in smallholder farmer fields in sub‐Saharan Africa are likely to experience water deficit alongside warmer heatwaves (Almazroui *et al*., 2020; Song *et al*., 2023). This combination can impact the ability of the plant to cool its leaves, leading to higher leaf temperatures (Carmo‐Silva *et al*., 2012; Fahad *et al*., 2017; Sato *et al*., 2024). Increasing the abundance of the superior Rca10α and Rca10β isoforms characterised here provides an exciting opportunity to enhance the resilience of cowpea and other crops to future extreme climates.

## Competing interests

None declared.

## Author contributions

EC‐S conceived and supervised the research. AG, RP, IR, DJO and EC‐S designed experiments. MTP designed and executed the cloning strategy. DB, PDG and DW produced and purified recombinant proteins. DW and CJA purified Rubisco. DB and AG performed ATP hydrolysis measurements. AG performed Rubisco reactivation measurements. RP and IR performed heatwave experiments. RP performed RNA‐seq, reverse transcription quantitative polymerase chain reaction and plant growth measurements. RP and PDG performed promoter analyses. IR performed fluorescence measurements. AG, RP and CJA determined the activity and amount of Rubisco and Rubisco activase in leaf extracts. AG, RP, CJA and IR analysed the data. AG and EC‐S wrote the manuscript with help from all authors.

## Disclaimer

The New Phytologist Foundation remains neutral with regard to jurisdictional claims in maps and in any institutional affiliations.

## Supporting information


**Fig. S1** Amino acid residue alignment of the four cowpea Rubisco activase isoforms.
**Fig. S2** *In vitro* Rubisco reactivation by the four cowpea Rubisco activase isoforms.
**Fig. S3** Heatwave experimental design and cabinet temperature monitoring.
**Fig. S4** Cabinet plant layout design.
**Fig. S5** Growth parameters and Chl content during and after heat stress.
**Fig. S6** Leaf temperature monitoring over the heatwave experiment.
**Fig. S7** *T*_crit_ calculation from temperature response of Chl fluorescence‐derived *F*_v_/*F*_m_.
**Fig. S8** Gene expression of heat shock protein 20 and cowpea Rubisco activase (reverse transcription quantitative polymerase chain reaction).
**Fig. S9** Protocol outline for determination of Rubisco activase activity in leaf extracts.
**Fig. S10** Specific Rubisco activities of control and heat‐treated plants.
**Fig. S11** Differential gene expression in heat‐treated vs control cowpea plants.
**Fig. S12** Volcano plots depicting differential gene expression in cowpea (*Vigna unguiculata* L.) plants based on log change across the days of heatwave.
**Fig. S13** Potential *cis*‐acting regulatory elements in cowpea Rubisco activase promoter regions.
**Fig. S14** Protein abundance of Rubisco activase isoforms in leaves of control and heat‐treated cowpea (*Vigna unguiculata* L.) plants.
**Fig. S15** Rubisco reactivation by the pool of cowpea (*Vigna* unguiculata L.) Rubisco activase isoforms in leaf extracts.
**Table S1** Reference protein sequences used to identify cowpea (*Vigna unguiculata* L.) Rubisco activase genes.
**Table S2** Primer sequences for adding Golden Gate overhangs to cowpea (*Vigna unguiculata* L.) Rubisco activase coding regions.
**Table S3** Modelling of the *in vitro* temperature response of ATP hydrolysis and Rubisco activation by cowpea (*Vigna unguiculata* L.) Rubisco activase isoforms.
**Table S4** Optimum temperature of *in vitro* Rubisco activase activity of cowpea (*Vigna unguiculata L*.) four isoforms.
**Table S5** RNA sample quality control (QC) analysis before RNA sequencing.
**Table S6** Sequencing and alignment statistics.
**Table S7** Minimum information for publication of quantitative real‐time PCR experiments (MIQE) checklist for reverse transcription quantitative polymerase chain reaction.
**Table S8** reverse transcription quantitative polymerase chain reaction primers.
**Table S9** Modelling of the temperature response of Rubisco activation by cowpea (*Vigna unguiculata* L.) Rubisco activase in leaf extracts of control and heat‐treated plants.
**Table S10** Leaf total soluble protein and Chl content *of* control and heat‐treated cowpea (*Vigna unguiculata* L.) plants.
Please note: Wiley is not responsible for the content or functionality of any Supporting Information supplied by the authors. Any queries (other than missing material) should be directed to the *New Phytologist* Central Office.

## Data Availability

Data presented are available in the accompanying supporting information and via Zenodo (doi: 10.5281/zenodo.15227676). The raw RNA‐seq data reported in this article have been deposited in DDBJ's Sequence Read Archive (DRA) and are accessible through DRR624522–DRR624545 under BioProject accession no. PRJDB19806. Metadata of RNA‐seq data are submitted in the DDBJ's Genomic Expression Archive (GEA) and are accessible through the Experiment accession no. E‐GEAD‐889.
