# Supplementary material for: Two cowpea Rubisco activase isoforms for crop thermotolerance
Source: New Phytol. 2025 Jun 8;247(3):1199–217. doi: 10.1111/nph.70271 (PMC12222919; doi:10.1111/nph.70271)
Supplement: Supplementary file 1 — Fig. S1 Amino acid residue alignment of the four cowpea Rubisco activase isoforms. Fig. S2 In vitro Rubisco reactivation by the four cowpea Rubisco activase isoforms. Fig. S3 Heatwave experimental design and cabinet temperature monitoring. Fig. S4 Cabinet plant layout design. Fig. S5 Growth parameters and Chl content during and after heat stress. Fig. S6 Leaf temperature monitoring over the heatwave experiment. Fig. S7 T crit calculation from temperature response of Chl fluorescence‐derived F v/F m. Fig. S8 Gene expression of heat shock protein 20 and cowpea Rubisco activase (reverse transcription quantitative polymerase chain reaction). Fig. S9 Protocol outline for determination of Rubisco activase activity in leaf extracts. Fig. S10 Specific Rubisco activities of control and heat‐treated plants. Fig. S11 Differential gene expression in heat‐treated vs control cowpea plants. Fig. S12 Volcano plots depicting differential gene expression in cowpea (Vigna unguiculata L.) plants based on log change across the days of heatwave. Fig. S13 Potential cis‐acting regulatory elements in cowpea Rubisco activase promoter regions. Fig. S14 Protein abundance of Rubisco activase isoforms in leaves of control and heat‐treated cowpea (Vigna unguiculata L.) plants. Fig. S15 Rubisco reactivation by the pool of cowpea (Vigna unguiculata L.) Rubisco activase isoforms in leaf extracts. Table S1 Reference protein sequences used to identify cowpea (Vigna unguiculata L.) Rubisco activase genes. Table S2 Primer sequences for adding Golden Gate overhangs to cowpea (Vigna unguiculata L.) Rubisco activase coding regions. Table S3 Modelling of the in vitro temperature response of ATP hydrolysis and Rubisco activation by cowpea (Vigna unguiculata L.) Rubisco activase isoforms. Table S4 Optimum temperature of in vitro Rubisco activase activity of cowpea (Vigna unguiculata L.) four isoforms. Table S5 RNA sample quality control (QC) analysis before RNA sequencing. Table S6 Sequencing and alignment st [file NPH-247-1199-s001.pdf]

New Phytologist Supporting Information

Article title: **Two cowpea Rubisco activase isoforms for crop thermotolerance**

Authors: Armida Gjindali; Rhiannon Page; Catherine J. Ashton; Ingrid Robertson; Mike T. Page; Duncan Bloemers; Peter D. Gould; Dawn Worrall, Douglas J. Orr; Elizabete Carmo-Silva

Lancaster Environment Centre, Lancaster University, Lancaster, LA1 4YQ, UK

Corresponding author email: e.carmosilva@lancaster.ac.uk

Article acceptance date: 12 May 2025

Supporting Information Figures 3

Fig. S1. Amino acid residue alignment of the four cowpea Rca isoforms. 3

Fig. S2. *In vitro* Rubisco reactivation by the four cowpea Rca isoforms. 4

Fig. S3. Experimental design and cabinet temperature monitoring. 5

Fig. S4. Cabinet plant layout design. 6

Fig. S5. Growth parameters and chlorophyll content during and after heat stress. 7

Fig. S6. Leaf temperature monitoring over the heatwave experiment. 8

Fig. S7. Method to calculate T_{crit} from chlorophyll fluorescence derived Fv/Fm over a temperature range. 9

Fig. S8. Gene expression of heat shock protein 20 (*HSP20*) and the four cowpea *Rca* transcripts. 11

Fig. S9. Protocol outline for determination of Rca activity in leaf extracts (LE). 12

Fig. S10. Specific Rubisco activities of control and heat-treated plants. 13

Fig. S11. Differential gene expression in heat-treated versus control cowpea plants. 14

Fig. S12. Volcano plots depicting differential gene expression in cowpea (*V. unguiculata* L.) plants based on log change across the days of heatwave. 15

Fig. S13. Identification of potential cis-acting regulatory elements in cowpea Rca promoter regions. 16

Fig. S14. Protein abundance of Rca isoforms in leaves of control and heat-treated cowpea (*V. unguiculata* L.) plants. 17

Fig. S15. Rubisco reactivation by the pool of cowpea (*V. unguiculata* L.) Rca isoforms in leaf extracts (LE). 18

Supporting Information Tables 19

Table S1. Reference protein sequences used to identify cowpea (*V. unguiculata* L.) Rca genes. 19

Table S2. Primer sequences for adding Golden Gate overhangs to cowpea. 20

Table S3. Modelling of the in vitro temperature response of ATP hydrolysis and Rubisco activation by cowpea (*V. unguiculata* L.) Rca isoforms. 21

Table S4. Optimum temperature of in vitro Rubisco activase (Rca) activity of cowpea (*V. unguiculata* L.) four isoforms. 22

Table S5. RNA sample QC analysis prior to RNA sequencing. 23

Table S6. Sequencing and alignment statistics. 24

Table S7. MIQE checklist for RT-qPCR. 25

Table S8. RT-qPCR primers. 27

Table S9. Modelling of the temperature response of Rubisco activation by cowpea (*V. unguiculata* L.) Rca in leaf extracts of control and heat-treat plants. 28

Table S10. Leaf total soluble protein (TSP) and chlorophyll content of control and heat-treated cowpea (*V. unguiculata* L.) plants. 29

References 30

Supporting Information Figures

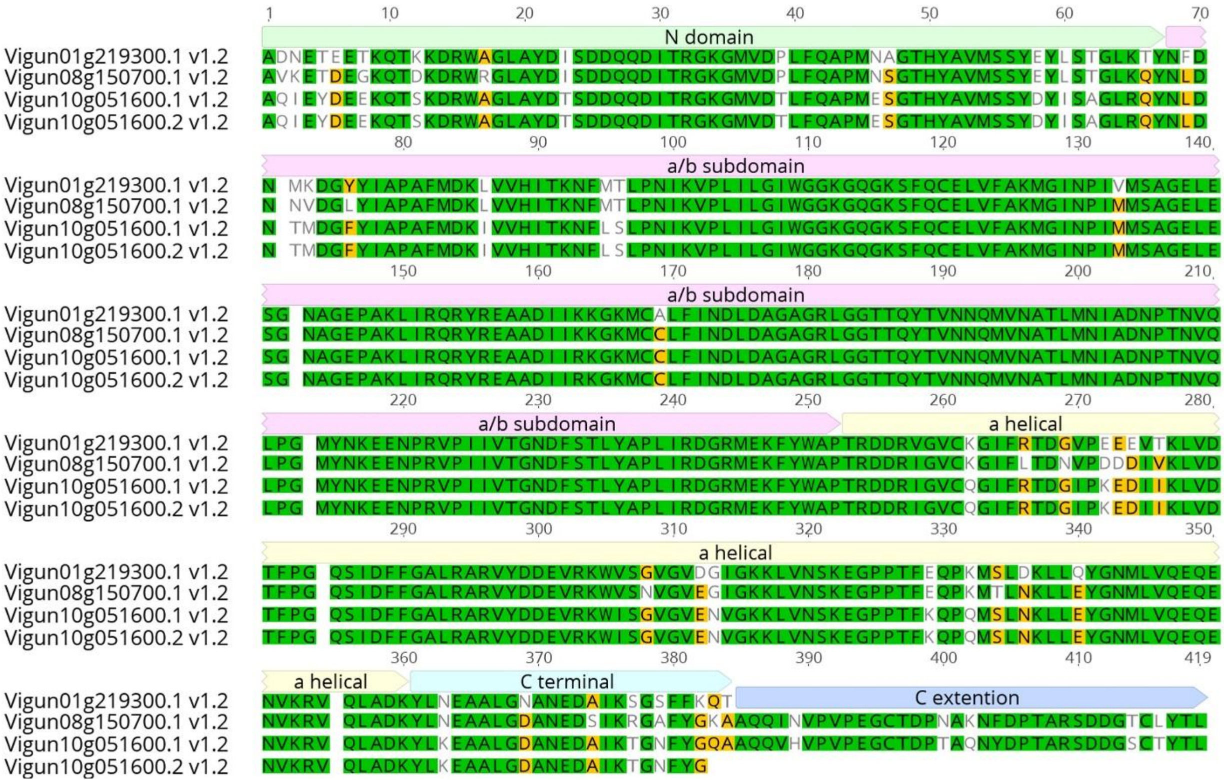

Fig. S1. Amino acid residue alignment of the four cowpea Rca isoforms. Shown are cowpea (*V. unguiculata* L.) mature protein sequences, excluding transit peptides. Rca1β (Vigun01g219300.1 v1.2), Rca8α (Vigun08g150700.1 v1.2), Rca10α (Vigun10g051600.1 v1.2), Rca10β (Vigun10g051600.2 v1.2). Annotations are based on structural domains of *N. tabacum* Rca (Stotz et al., 2011). Sequences were retrieved from Phytozome 13 and the alignment was generated in Geneious 9.1.8.

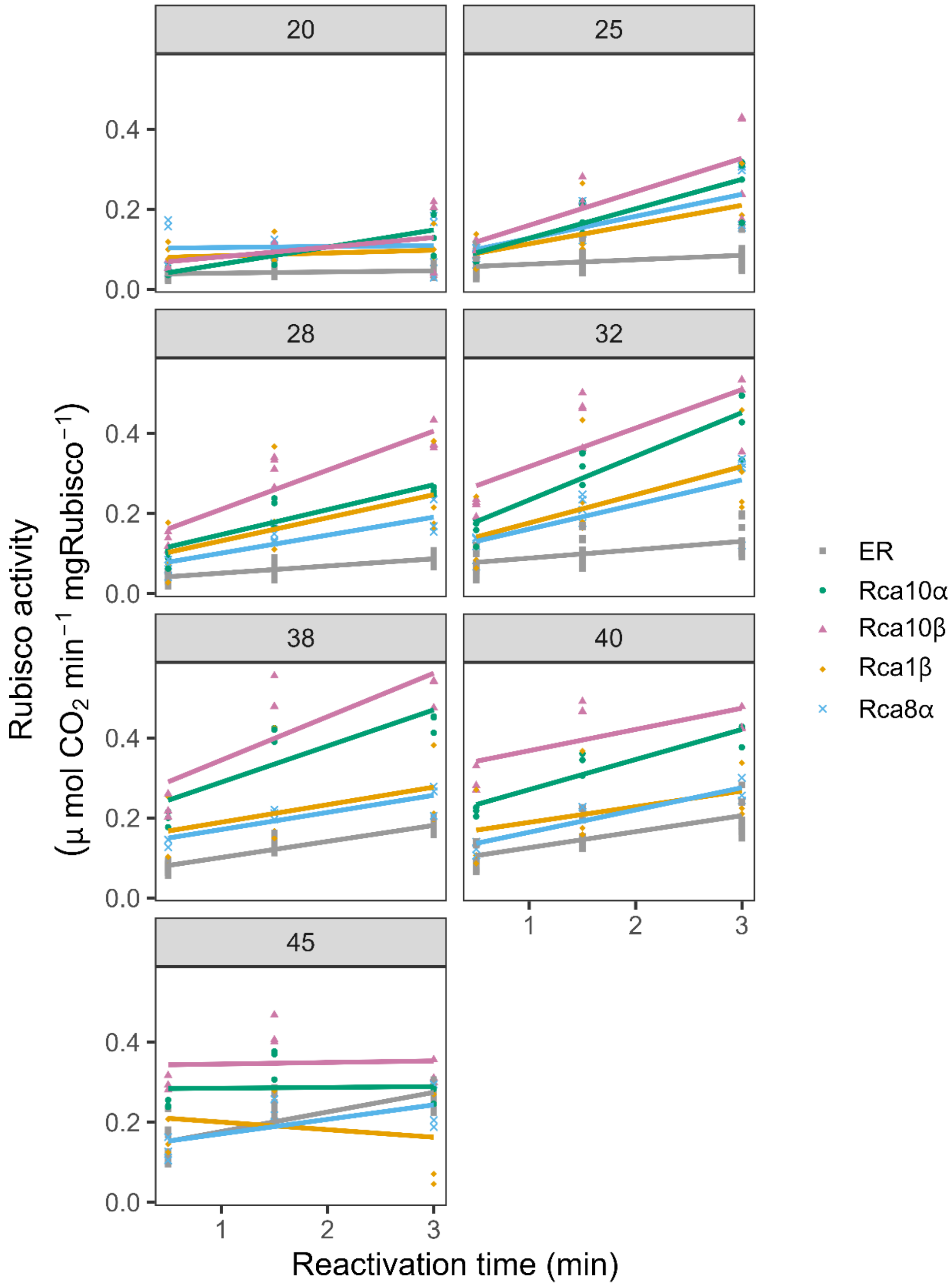

Fig. S2. ***In vitro*** **Rubisco reactivation by the four cowpea Rca isoforms.** a) Spontaneous (ER) and Rca mediated reactivation of Rubisco at different timepoints of temperature incubation of the four cowpea (*V. unguiculata* L.) isoforms Rca1β, Rca8α, Rca10α, Rca10β. For each temperature and each timepoint four technical replicates of ER were performed and one of each Rca isoform. Symbols represent biological replicates (independent protein purifications). In total for each isoform, activity was determined in three biological replicates (unique purifications) for temperatures 20-32℃ and four for temperatures 38-45℃ (ER n=18, Rca n=3-4).

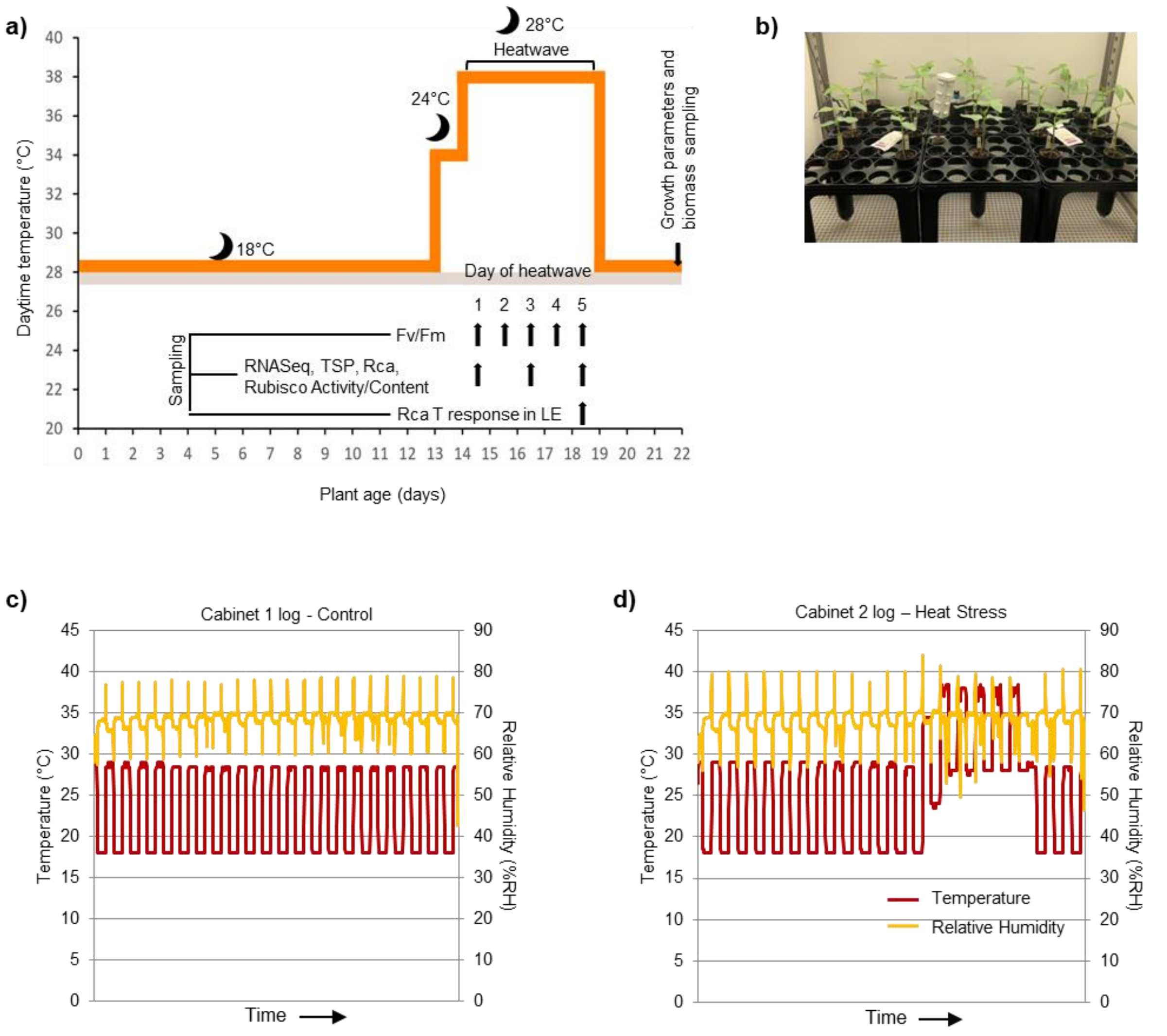

Fig. S3. Experimental design and cabinet temperature monitoring. a) Overview of the treatments applied to the cowpea (*V. unguiculata* L.) plants, where orange = temperatures of heat-treated plants, and grey = temperature of control plants. Night temperatures for each part of the study are shown next to moon symbols. Time of sampling for various analyses is shown by black arrows. b) Picture of plants setup and data logger position. Environmental conditions, air temperature and relative humidity, in a representative control cabinet (c) and heat stress cabinet (d).

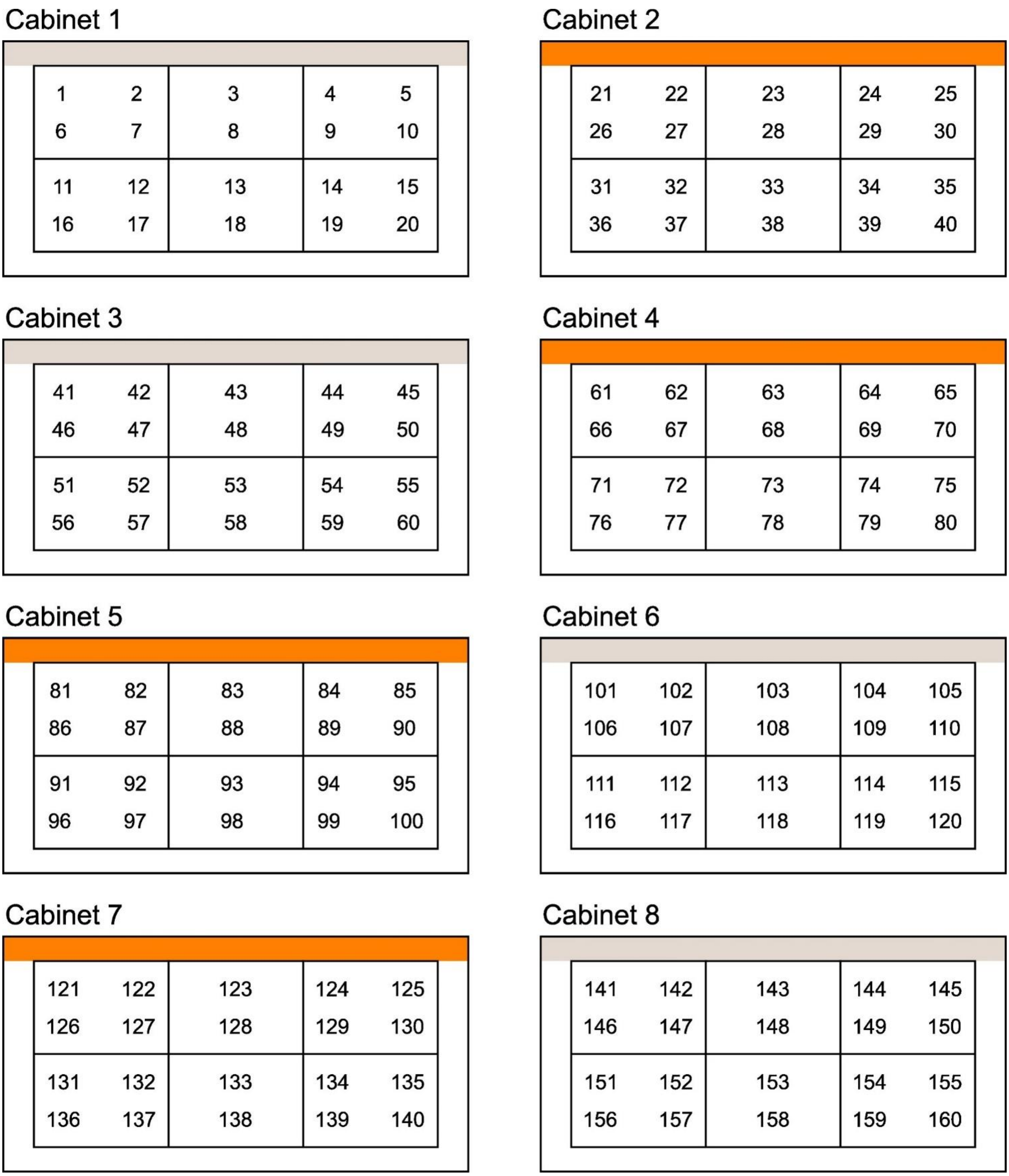

Fig. S4. Cabinet plant layout design. Plants were distributed among eight cabinets: four maintained at control temperatures (grey) throughout the experiment and four subjected to a heatwave (orange).

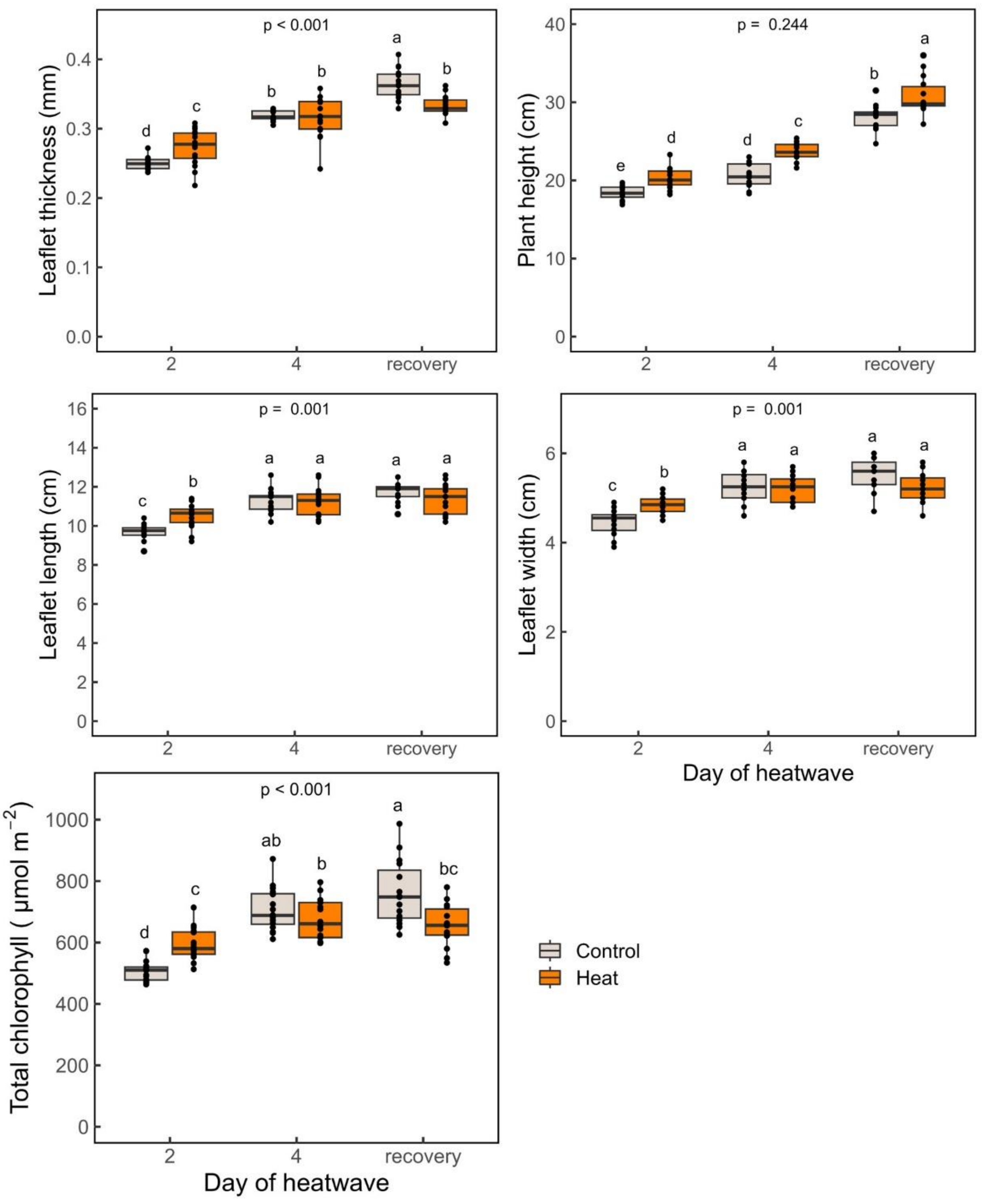

Fig. S5. Growth parameters and chlorophyll content during and after heat stress. Parameters were measured in cowpea (*V. unguiculata* L.) control and heat-treated plants on days 2 and 4 of the heatwave. Recovery corresponds to four days after the end of the heatwave where temperature is reversed back to control conditions (day 22 of growth). Chlorophyll content was measured using a handheld meter. Box plots show medians and the first and third quartiles (25th and 75th percentiles), and whiskers extend from the hinge to the largest or smallest value. Symbols represent individual data points (biological replicates). Interaction p-values were determined using a two-way ANOVA followed by Tukey's post-hoc test (n=15-16).

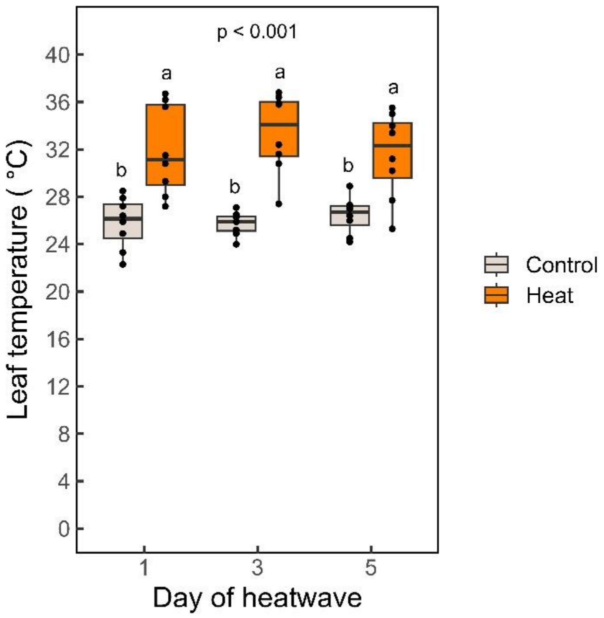

Fig. S6. Leaf temperature monitoring over the heatwave experiment. Leaf temperature was measured in control and heat-treated cowpea (*V. unguiculata* L.) with a thermal camera on days 1, 3 and 5 of the heatwave, just before taking leaf samples for analysis. There was no significant interaction between days and treatment (p=0.435), the treatment p-value shown was determined using a two-way ANOVA followed by Tukey's post-hoc test (n= 8). Box plots show medians and the first and third quartiles (25th and 75th percentiles), and whiskers extend from the hinge to the largest or smallest value. Symbols represent individual data points (biological replicates). The mean leaf temperature across all days for control plants was 26 ± 0.3 °C and for heat-treated plants 32 ± 0.7 °C.

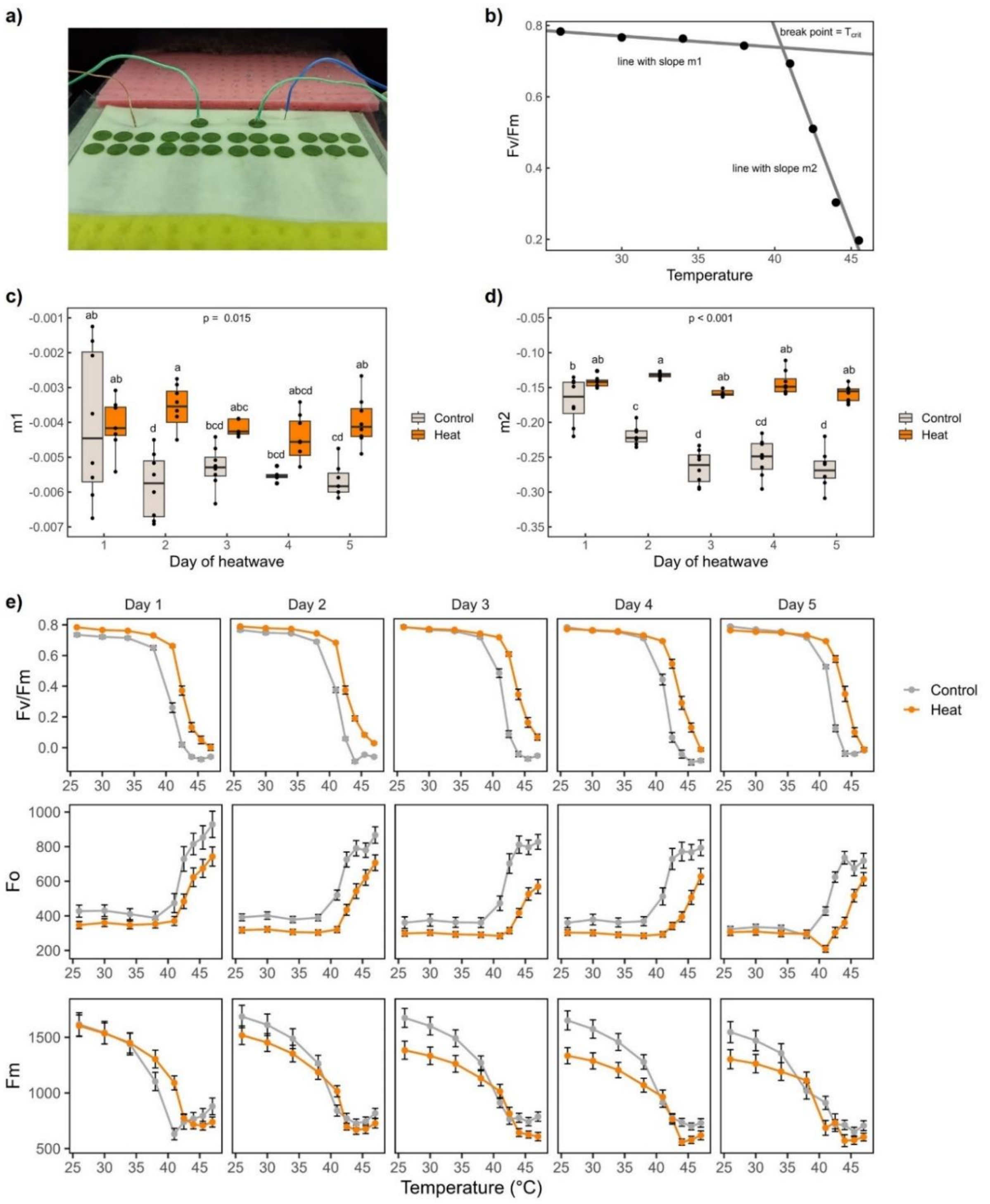

Fig. S7. Method to calculate T_{crit} from chlorophyll fluorescence derived Fv/Fm over a temperature range. a) Layout of cowpea (*V. unguiculata* L.) leaf discs in fluorescence imager showing sample surface with wet filter paper, sponges to provide reservoir of water and thermocouple placement. b) Single example of Fv/Fm values over a temperature range showing the fitted straight lines with slopes m_1 & m_2 and the breakpoint T_{crit}. c) m_1 (n=6-7). d) m_2 (n=7-8). Box plots show medians and the first and third quartiles (25th and 75th percentiles), and whiskers extend from the hinge to the largest or smallest value. Interaction p-values were determined using a two-way ANOVA followed by Tukey's post-hoc test. e) Fv/Fm, Fo and Fm

over the temperature range and across the five days of heatwave. Points show the treatment averages and error bars show standard errors (n=8).

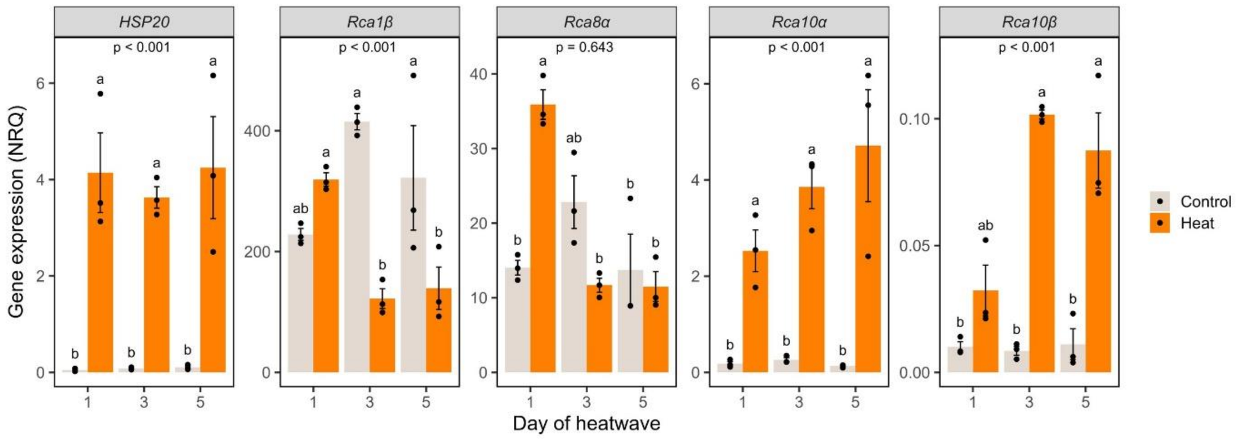

Fig. S8. Gene expression of heat shock protein 20 (*HSP20*) and the four cowpea *Rca* transcripts. RT-qPCR was performed on cowpea (*V. unguiculata* L.) leaf disc samples taken at days 1,3, and 5 of the heatwave, in a replicate experiment using the same design as the RNA-seq analyses (Fig. 2a). RNA extraction and RT-qPCR analysis information is provided in the MIQE checklist (Table) and primer sequences and information in Supplementary Table 8. Key results are confirmatory of the RNA-seq analysis, showing a significant increase in gene expression of *Rca10α* and *Rca10β* during the heatwave. Bar plots show means and error bars represent standard error. Symbols represent individual data points (biological replicates). Two-way ANOVA followed by Tukey's post-hoc test was performed after log transformation (n=3). P-values correspond to heat treatment effect across the days of the heatwave for each of the genes.

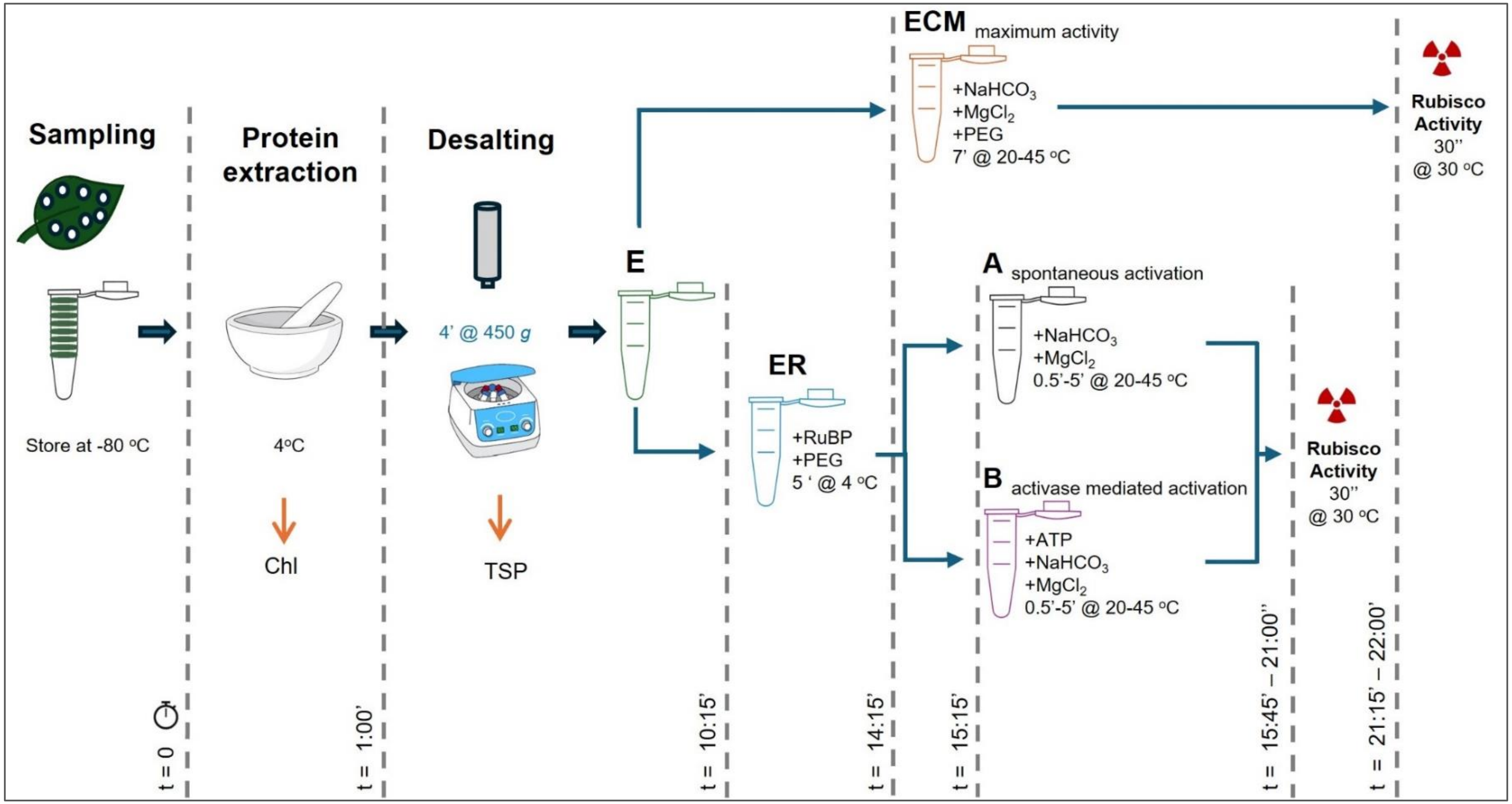

Fig. S9. Protocol outline for determination of Rca activity in leaf extracts (LE). Samples consisting of eight leaf discs per plant were collected on day 5 of the heatwave. The protocol timeline starts (t=0) with protein extraction by grinding leaves in ice-cold extraction buffer. Homogenate aliquots were used for leaf chlorophyll content determination. A spin desalting step enables removal of Mg^{2+} ions and CO_2 to obtain free uncarbarmylated Rubisco (E). Aliquots of the desalted leaf extract were taken for total soluble protein (TSP), activity of fully carbamylated Rubisco (ECM) and inhibition of uncarbamylated Rubisco by binding to RuBP (ER) for Rca activity determination. The ECM sample was incubated with 5% PEG3350, 10 mM $NaHCO_3$, and 30 mM $MgCl_2$ for 7 min at 20-45 °C prior to measuring Rubisco activity at 30 °C. The ER sample was supplemented with 5% PEG3350 and 4 mM RuBP and incubated for 5 min at 4 °C to form the inhibited Rubisco–RuBP (ER) complex. The leaf extract containing ER and Rca was then used to initiate reactivation assays with 10 mM $NaHCO_3$ and 30 mM $MgCl_2$ in the presence or absence of 5 mM ATP plus an ATP-regenerating system at 20-45 °C to determine spontaneous (A) and activase-mediated (B) activation of Rubisco by measuring the increase in Rubisco activity at 30 °C.

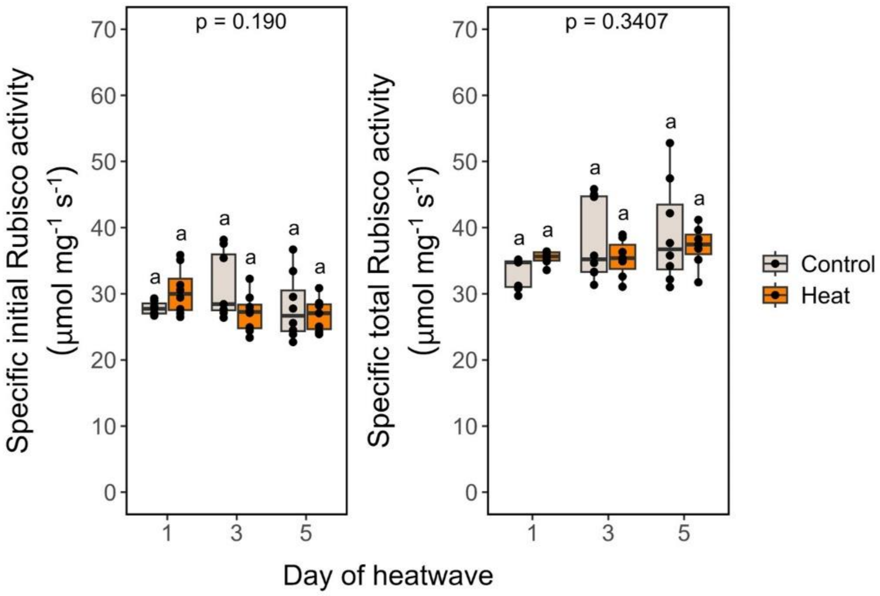

Fig. S10. Specific Rubisco activities of control and heat-treated plants. Initial and total activities of cowpea (*V. unguiculata* L.) control and heat-treated plants were normalised to the Rubisco concentration. Box plots show medians and the first and third quartiles (25th and 75th percentiles), and whiskers extend from the hinge to the largest or smallest value. Symbols represent individual data points (biological replicates). Interaction p-values were determined using a two-way ANOVA followed by Tukey's post-hoc test (n=6-8).

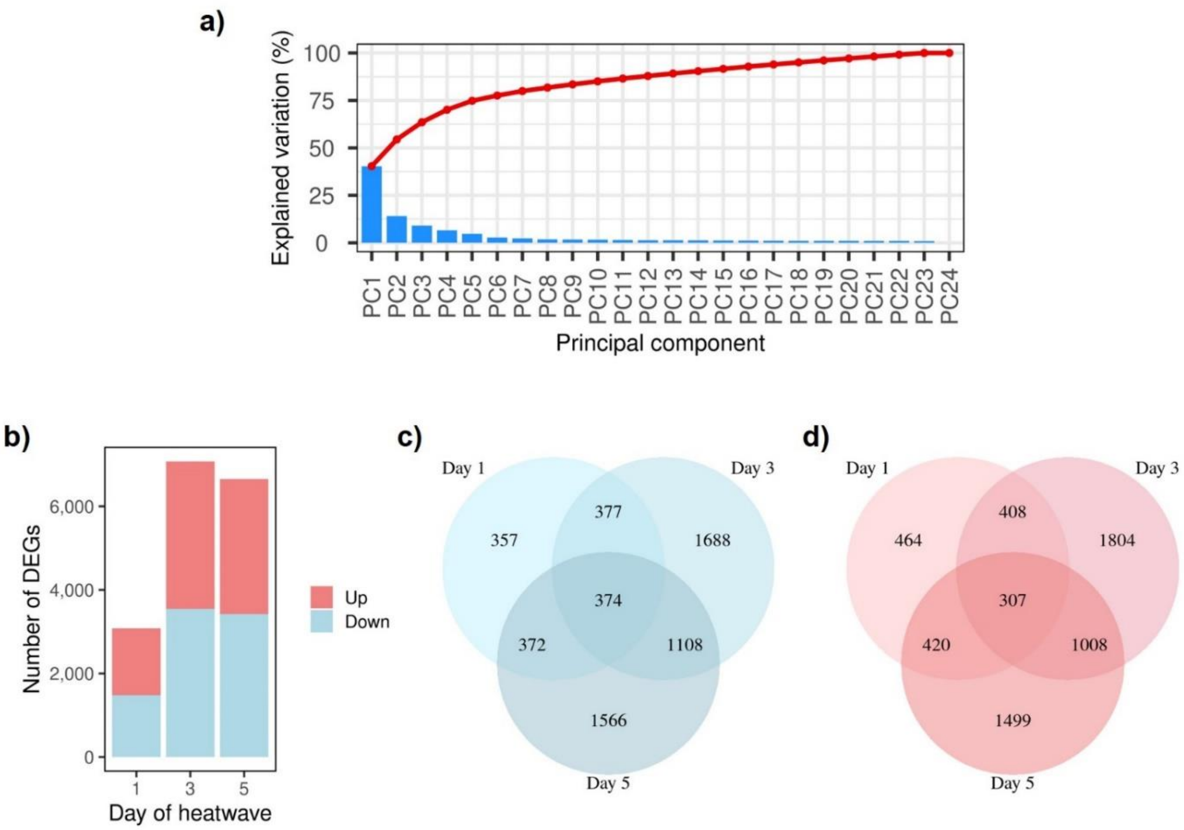

Fig. S11. Differential gene expression in heat-treated versus control cowpea plants. Young cowpea (*V. unguiculata* L.) plants were exposed to a +10 °C heatwave for 5 days and samples taken for RNA-seq analysis on days 1, 3, 5. a) Scree plot showing the principal components (PCA) driving differentially expressed genes (DEGs) in control and heat-treated plants. b) The number of DEGs that are downregulated and upregulated at days 1, 3, and 5 of the heat treatment. (c, d) Venn diagrams showing the number of unique and common downregulated (c) and upregulated (d) DEGs in heat-treated compared to control plants on days 1, 3 and 5. Venn diagrams were produced using the VennDiagram in R studio.

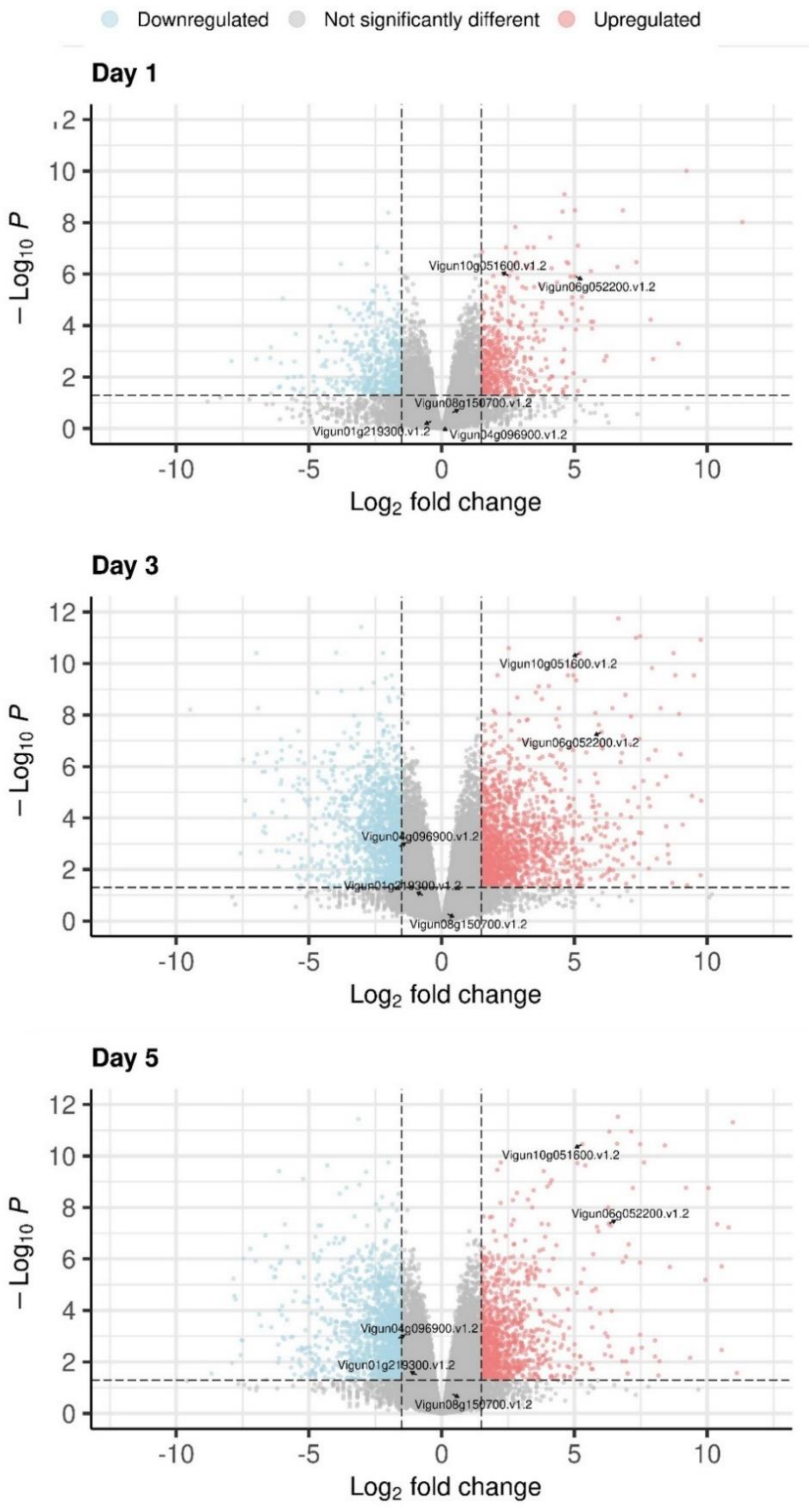

Fig. S12. Volcano plots depicting differential gene expression in cowpea (*V. unguiculata* L.) plants based on log change across the days of heatwave. Blue corresponds to the downregulated genes, coral red to upregulated gene expression with positive log fold change during the heatwave while middle grey corresponds to the genes whose expression was unaffected by the heat treatment. Labels correspond to the *HSP20* (*Vigun06g052200*.v1.2), *RbcS* (*Vigun04g096900*.v1.2) and *Rca* encoding genes (*Rca1*: *Vigun01g219300*.v1.2, *Rca8*: *Vigun08g150700*.v1.2, *Rca10*: *Vigun10g051600*.v1.2).

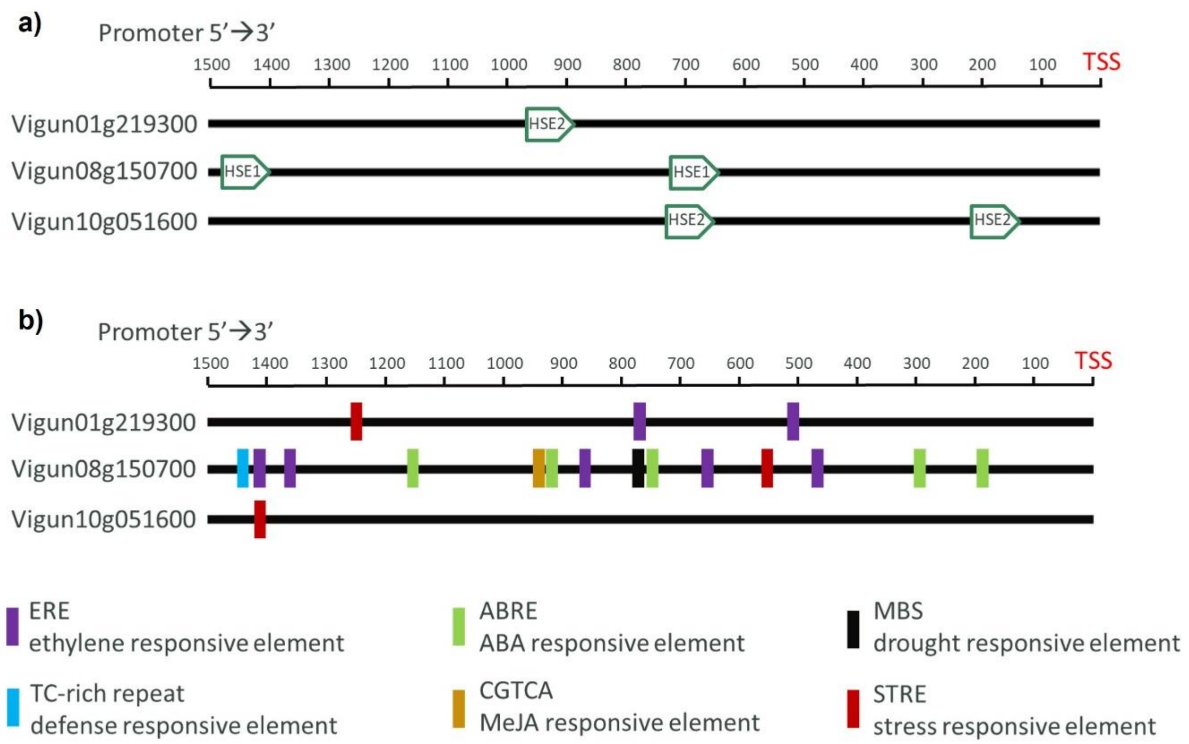

c)

Cis Regulatory Motif	VuRca1	VuRca8	VuRca10	Signal Seq	Function
TATABOXOSPAL		2	3	TATTTAA	BIOSYNTHESIS, DEFENCE, DROUGHT STRESS
WBOXNTERF3	1	2	3	TGACY	DEFENCE, STRESS
GT1GMSCAM4	6	6	6	GAAAAA	DEFENCE, STRESS
PRECONSCRHSP70A	1			SCGAYNRNNNNNNNNNNNNNNNHD	LIGHT/ STRESS?
MYB1AT	4	3		WAACCA	STRESS
MYCCONSENSUSAT	6	14		CANNTG	STRESS
MYB2CONSENSUSAT		2		YAACKG	STRESS
BIHD1OS	2	8	2	TGTCA	STRESS
TATABOX3	2	2	2	TATTAAT	STRESS
MYBCORE	1	2		CNGTTR	STRESS
ABREZMRAB28	2			CCACGTGG	STRESS
EMBP1TAEM	1			CACGTGGC	STRESS
IRO2OS	2			CACGTGG	STRESS
ABREATCONSENSUS	1			YACGTGGC	STRESS
ACGTATERD1	4	8	2	ACGT	STRESS
MYB2AT		1		TAACTG	STRESS
MYCATERD1	1	3		CATGTG	STRESS
MYCATRD22	1	3		CACATG	STRESS
CURECORECR	2	2	4	GTAC	STRESS, COPPER DEFICIENCY
CCAATBOX1	8	5	2	CCAAT	STRESS, HEAT SHOCK, TEMPERATURE
ABRERATCAL	2	2		MACGYGB	STRESS, HORMONE
ACGTABREMOTIFA2OSEM	1	2		ACGTGKC	STRESS, HORMONE
ABRELATERD1	1	5		ACGTG	STRESS, HORMONE
SURECOREATSULTR11	5		3	GAGAC	SULPHUR DEFICIENCY, STRESS, HORMONE
LTRECOREATCOR15	1			CCGAC	TEMP, LIGHT
CBFHV			2	RYCGAC	TEMPERATURE
CRTDREHVCBF2			2	GTCGAC	TEMPERATURE

Fig. S13. Identification of potential cis-acting regulatory elements in cowpea Rca promoter regions. a) To identify potential cis-acting regulatory elements in cowpea (*V. unguiculata* L.) *Rca* promoter regions, 1.5kb regions upstream of the transcriptional start sites (TSSs) were investigated for heat stress elements (HSE) based on consensus sequences identified by Jung *et al.* (2013). Several instances of consensus HSE sequences HSE1 (GAAnnTTC) and HSE2 (TTCnnGAA) were identified. The same promoter regions were analysed using PlantCARE (b) and PLACE (c) to determine the number of motifs related to temperature and stress response.

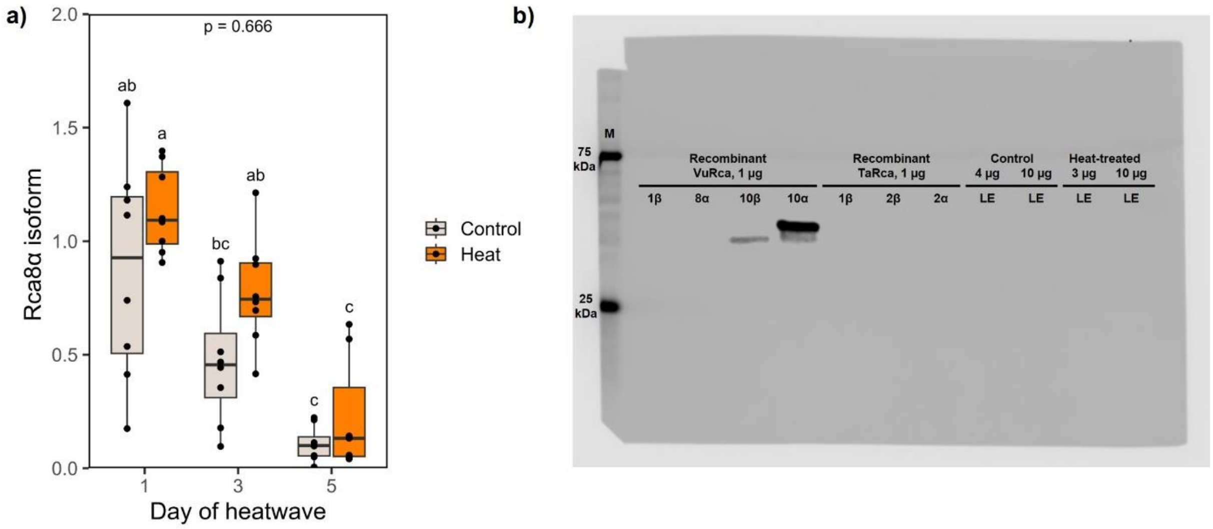

Fig. S14. Protein abundance of Rca isoforms in leaves of control and heat-treated cowpea (*V. unguiculata* L.) plants. a) A specific antibody that reacts only with Rca8α (Bloemers & Carmo-Silva, 2024) was used to quantify the abundance of this protein isoform. Anti-Rca8α antibody was generated using phage display where the selected peptide targeted residues 376-391 of the mature protein sequence (KRGAFYGKAAQQINVP)(Bloemers & Carmo-Silva, 2024). Blots were analysed as described in Methods. Box plots show medians and the first and third quartiles (25th and 75th percentiles), and whiskers extend from the hinge to the largest or smallest value. Symbols represent individual data points (biological replicates). Interaction p-values were calculated using two-way ANOVA followed by Tukey post-hoc test (n=7-8). b) An anti-Rca10α/β antibody was also generated using phage display, where the selected peptide targeted residues 376-391 of the mature protein sequence (KTGNFYGQAAQQVHVP). The anti-Rca10α/β antibody showed detection of recombinant VuRca10, however, no visible bands were seen for control and heat-treated leaf extract (LE) samples. Both the leaf extract samples tested were sampled on day 5 of the heatwave. There was also no binding of the anti-Rca10α/β antibody to recombinant wheat Rca (TaRca).

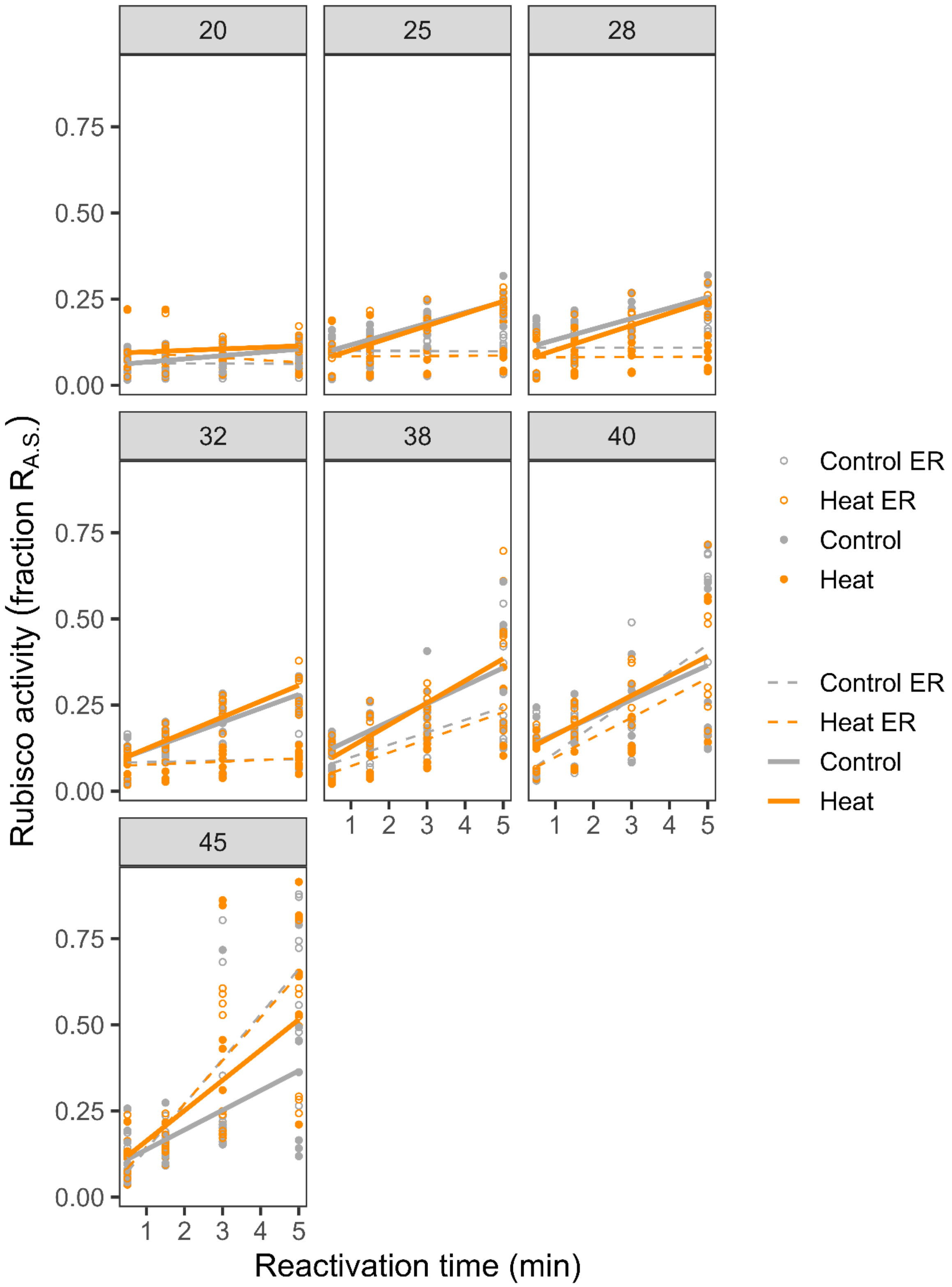

Fig. S15. Rubisco reactivation by the pool of cowpea (*V. unguiculata* L.) Rca isoforms in leaf extracts (LE). Spontaneous (open circles, dashed line) and Rca mediated (filled circles, solid line) reactivation of Rubisco at different timepoints of temperature incubation of control (grey) and heat-treated (orange) plants (n=7-8). Samples were collected on day 5 of the heatwave. Symbols represent individual measurements.

Supporting Information Tables

Table S1. Reference protein sequences used to identify cowpea (*V. unguiculata* L.) Rca genes.

Species	Protein Name	Identifier (Database)
Arabidopsis thaliana	AtRca	At2g39730 (TAIR)
Nicotiana tabacum	NtRca	Q40460 (Uniprot)
Triticum aestivum	TaRca1	TraesCS4A02G177600 (EnsemblPlants)
	TaRca2	TraesCS4A02G177500 (EnsemblPlants)

Table S2. Primer sequences for adding Golden Gate overhangs to cowpea (*V. unguiculata* L.) Rca coding regions. For each Rca coding sequence a specific primer pair was used, except for Rca10β where the forward primer for Rca10α (Rca10.1A_GG_F) could be used due to the identical coding sequence at the 5’-end.

Primer Name	Sequence (5’ > 3’)	Notes
Rca1.1B_GG_F	GCGGTCTCACAATGCCGACAACGAAACGGAG	Adds Golden Gate sites to Rca1
Rca1.1B_GG_R	CAGGTCTCGATCATTAAGTCTGTTTGAAGAAGC	
Rca8.1A_GG_F	GCGGTCTCACAATGCAGTCAAAGAAACCGATG	Adds Golden Gate sites to Rca8
Rca8.1A_GG_R	CAGGTCTCGATCATCACAGAGTGTACAGGCAC	
Rca10.1A_GG_F	GCGGTCTCACAATGCGCAGATCGAATATGACG	Adds Golden Gate sites to Rca10a
Rca10.1A_GG_R	CAGGTCTCGATCACTACAGGGTGTACGTGCAG	
Rca10.2B_GG_R	CAGGTCTCGATCATCAACCGTAGAAGTTACCGGT	Adds Golden Gate site to Rca10b

Table S3. Modelling of the *in vitro* temperature response of ATP hydrolysis and Rubisco activation by cowpea (*V. unguiculata* L.) Rca isoforms. The model providing the best fit to the data is highlighted in bold and was selected according to the lowest AIC score (Akaike information criterion) calculated according to Akaike (1974) using the AIC function in R. Models were applied to the full dataset shown in Fig. 1b (n=3-4).

Rca Isoform	ATPase assay			Rubisco activation assay		
	Model	degrees of freedom (K)	AIC score	Model	degrees of freedom (K)	AIC score
1β	2nd order poly	4	218	2nd order poly	4	-16
	3rd order poly	5	219	3rd order poly	5	-15
	**4th order poly**	**6**	**211**	**4th order poly**	**6**	**-17**
	5th order poly	7	213	5th order poly	7	-15
	GAM	3	237	GAM	3	-14
8α	**2nd order poly**	**4**	**176**	2nd order poly	4	-16
	3rd order poly	5	177	**3rd order poly**	**5**	**-18**
	4th order poly	6	178	4th order poly	6	-16
	5th order poly	7	179	5th order poly	7	-14
	GAM	3	201	GAM	3	-11
10α	2nd order poly	4	357	**2nd order poly**	**4**	**-24**
	**3rd order poly**	**5**	**356**	3rd order poly	5	-22
	4th order poly	6	358	4th order poly	6	-21
	5th order poly	7	360	5th order poly	7	-24
	GAM	3	373	GAM	3	-0.7
10β	**2nd order poly**	**4**	**368**	2nd order poly	4	-26
	3rd order poly	5	369	**3rd order poly**	**5**	**-28**
	4th order poly	6	371	4th order poly	6	-26
	5th order poly	7	372	5th order poly	7	-28
	GAM	3	376	GAM	3	8

Table S4. Optimum temperature of *in vitro* Rubisco activase (Rca) activity of cowpea (*V. unguiculata* L.). Maximum rates of ATP hydrolysis and Rubisco reactivation, and corresponding temperature of maximum activity (T_{max}), optimum temperature range (T_{opt}, above 70% activity) and temperature above the optimum at which 50% of the maximum activity remains ($T_{0.5}$), as estimated from the best-fit models applied to describe the *in vitro* temperature response of each Rca isoform (Table S). The best fitting model was applied to the combined biological replicates presented in Fig. 1b (n=3-4).

**Rca Isoform**	**$ATPase_{max}$ (mol min^{-1} mol Rca^{-1})**	**T_{max} (°C)**	**T_{opt} (°C)**	**$T_{0.5}$ (°C)**
**1β**	27.4	36.4	30.2 – 42.4	44.4
**8α**	35.3	35.9	25.7 – 46	49.0
**10α**	192.1	42.3	31.4 – 50.9	53.1
**10β**	181.3	39.3	28.0 – 50.7	54.1
**Rca Isoform**	**Rubisco $reactivation_{max}$ (mol $R_{A.S.}$ min^{-1} mol Rca^{-1})**	**T_{max} (°C)**	**T_{opt} (°C)**	**$T_{0.5}$ (°C)**
**1β**	0.37	29.9	25.9 – 35	37.1
**8α**	0.33	27.8	22.9 – 33.9	36.4
**10α**	0.54	32.3	25.6 – 40.3	42.0
**10β**	0.74	34.4	26.7 – 40.9	42.6

Table S5. RNA sample QC analysis prior to RNA sequencing.

			MICROPLATE READER			NOVOGENE			
**Sample ID**	**Treatment**	**Timepoint**	**Concentration (ng/µl)**	**260/280 ratio**	**260/230 ratio**	**Concentration (ng/µl)**	**Volume (µl)**	**Total amount (µg)**	**Integrity value (RIN)**
RP001	Control	D5	311.75	2.01	2.31	236.46	41	9.69	7.1
RP002	Control	D1	284.21	2.01	2.40	228.61	42	9.60	8.3
RP020	Control	D3	725.36	2.05	2.39	866.62	42	36.40	8.8
RP031	Heat	D1	488.38	2.02	2.38	529.17	42	22.23	8.5
RP038	Heat	D5	80.82	1.98	2.29	50.29	42	2.11	7.7
RP040	Heat	D3	103.86	1.99	2.16	70.71	42	2.97	8.0
RP051	Control	D5	321.12	2.01	2.40	240.84	42	10.12	7.4
RP058	Control	D1	481.18	2.03	2.38	559.28	42	23.49	9.0
RP059	Control	D3	340.80	2.02	2.27	282.79	38	10.75	7.6
RP070	Heat	D5	121.95	2.00	2.19	86.02	42	3.61	7.3
RP077	Heat	D1	785.70	2.05	2.39	1005.5	42	42.23	8.5
RP078	Heat	D3	192.73	2.00	2.33	130.67	42	5.49	7.9
RP086	Heat	D5	144.70	1.98	2.22	99.35	42	4.17	7.7
RP092	Heat	D1	582.69	2.03	2.39	702.46	42	29.50	8.9
RP098	Heat	D3	293.68	2.01	2.37	226.71	42	9.52	7.8
RP111	Control	D3	519.96	2.03	2.32	612.69	42	25.73	8.8
RP115	Control	D5	144.46	2.00	2.29	105.08	38	3.99	6.8
RP120	Control	D1	458.21	2.02	2.38	499.47	41	20.48	8.9
RP121	Heat	D5	133.62	1.99	2.24	86.53	43	3.72	6.3
RP122	Heat	D3	236.64	2.00	2.38	171.98	42	7.22	8.2
RP130	Heat	D1	764.49	2.05	2.40	977.39	42	41.05	8.7
RP141	Control	D3	600.24	2.04	2.40	659.07	41	27.02	8.6
RP144	Control	D5	137.09	2.00	2.24	93.48	40	3.74	7.7
RP159	Control	D1	698.14	2.05	2.40	802.96	40	32.12	8.9

Table S6. Sequencing and alignment statistics.

Sample ID	Treatment	Timepoint	Data_file_size (bytes)	Number_seq_raw	Number_seq_clean	Number_seq_removed	Percentage_seq_removed	Total_bases_raw (Gbp)	Total_bases_clean (Gbp)	GC_content (%)	Mean_quality_per_read (Phred score)
RP001_1	control	D5	13425721087	36045152	35537602	507550	1.41	5.4	5.2	46	36
RP001_2	control	D5	13425721087	36045152	35537602	507550	1.41	5.4	5.2	46	36
RP002_1	control	D1	11605151548	31157275	30748411	408864	1.31	4.6	4.5	46	36
RP002_2	control	D1	11605151548	31157275	30748411	408864	1.31	4.6	4.5	46	36
RP020_1	control	D3	12056176450	32368235	31981933	386302	1.19	4.8	4.7	46	36
RP020_2	control	D3	12056176450	32368235	31981933	386302	1.19	4.8	4.7	46	36
RP031_1	heat	D1	13190424154	35413441	34930680	482761	1.36	5.3	5.1	46	36
RP031_2	heat	D1	13190424154	35413441	34930680	482761	1.36	5.3	5.1	46	36
RP038_1	heat	D5	12819613510	34417863	33977748	440115	1.28	5.1	5.0	44	36
RP038_2	heat	D5	12819613510	34417863	33977748	440115	1.28	5.1	5.0	44	36
RP040_1	heat	D3	14684686925	39425215	38884497	540718	1.37	5.9	5.7	45	36
RP040_2	heat	D3	14684686925	39425215	38884497	540718	1.37	5.9	5.7	45	36
RP051_1	control	D5	15268299964	40992041	40445324	546717	1.33	6.1	6.0	46	36
RP051_2	control	D5	15268299964	40992041	40445324	546717	1.33	6.1	6.0	46	36
RP058_1	control	D1	17162706419	46078190	45422671	655519	1.42	6.9	6.7	46	36
RP058_2	control	D1	17162706419	46078190	45422671	655519	1.42	6.9	6.7	46	36
RP059_1	control	D3	14002141251	37592419	37121062	471357	1.25	5.6	5.5	46	36
RP059_2	control	D3	14002141251	37592419	37121062	471357	1.25	5.6	5.5	46	36
RP070_1	heat	D5	13799927700	37049814	36573118	476696	1.29	5.5	5.4	44	36
RP070_2	heat	D5	13799927700	37049814	36573118	476696	1.29	5.5	5.4	44	36
RP077_1	heat	D1	12530825305	33642540	33192110	450430	1.34	5.0	4.9	46	36
RP077_2	heat	D1	12530825305	33642540	33192110	450430	1.34	5.0	4.9	46	36
RP078_1	heat	D3	11027898150	29607379	29210364	397015	1.34	4.4	4.3	45	36
RP078_2	heat	D3	11027898150	29607379	29210364	397015	1.34	4.4	4.3	45	36
RP086_1	heat	D5	11671673556	31335849	30838307	497542	1.59	4.7	4.5	45	36
RP086_2	heat	D5	11671673556	31335849	30838307	497542	1.59	4.7	4.5	45	36
RP092_1	heat	D1	12620133920	33882356	33446049	436307	1.29	5.0	4.9	46	36
RP092_2	heat	D1	12620133920	33882356	33446049	436307	1.29	5.0	4.9	46	36
RP098_1	heat	D3	11830130671	31761357	31313377	447980	1.41	4.7	4.6	45	36
RP098_2	heat	D3	11830130671	31761357	31313377	447980	1.41	4.7	4.6	45	36
RP111_1	control	D3	13624866297	36579818	36114521	465297	1.27	5.4	5.3	46	36
RP111_2	control	D3	13624866297	36579818	36114521	465297	1.27	5.4	5.3	46	36
RP115_1	control	D5	13725270653	36849316	36385324	463992	1.26	5.5	5.4	45	36
RP115_2	control	D5	13725270653	36849316	36385324	463992	1.26	5.5	5.4	45	36
RP120_1	control	D1	14283643262	38348422	37812563	535859	1.40	5.7	5.6	46	36
RP120_2	control	D1	14283643262	38348422	37812563	535859	1.40	5.7	5.6	46	36
RP121_1	heat	D5	13625006123	36580213	36043152	537061	1.47	5.4	5.3	44	36
RP121_2	heat	D5	13625006123	36580213	36043152	537061	1.47	5.4	5.3	44	36
RP122_1	heat	D3	16076158335	43161019	42625226	535793	1.24	6.4	6.3	45	36
RP122_2	heat	D3	16076158335	43161019	42625226	535793	1.24	6.4	6.3	45	36
RP130_1	heat	D1	13614565840	36552173	36044664	507509	1.39	5.4	5.3	46	36
RP130_2	heat	D1	13614565840	36552173	36044664	507509	1.39	5.4	5.3	46	36
RP141_1	control	D3	12326927737	33095161	32575078	520083	1.57	4.9	4.8	46	36
RP141_2	control	D3	12326927737	33095161	32575078	520083	1.57	4.9	4.8	46	36
RP144_1	control	D5	11949656862	32082287	31624594	457693	1.43	4.8	4.7	45	36
RP144_2	control	D5	11949656862	32082287	31624594	457693	1.43	4.8	4.7	45	36
RP159_1	control	D1	11311705486	30369485	29954078	415407	1.37	4.5	4.4	46	36
RP159_1	control	D1	11311705486	30369485	29954078	415407	1.37	4.5	4.4	46	36

Table S7. MIQE checklist for RT-qPCR. Plants for RT-qPCR were grown alongside plants for RNA-seq analysis. Leaf discs were collected from 3 independent biological replicates per treatment per timepoint (days 1, 3 and 5 of heatwave) similarly to RNA-seq sampling (Methods). Detailed information for RNA extraction and RT-qPCR analysis are listed below in accordance with the MIQE guidelines. For primer sequences and information seeTable .

**MIQE checklist** (as per Bustin *et al.* (2009))	
**Experimental design**	
Definition of experimental and control groups	Experimental group: cowpea (*V. unguiculata* L.) plants exposed to heat treatment<br>Control group: cowpea plants grown under control conditions
Number within group	3 independent biological replicates per group, each grown in different cabinets. Each cabinet contained a separate plant for each timepoint. 3 technical replicates were performed for RT-qPCR.
**Sample**	
Description	Cowpea leaf material
Processing	0.55 cm^2 cork borer used to cut leaf discs, immediately snap frozen in liquid nitrogen, stored at -80 °C for <6 months before RNA extraction
**Nucleic acid extraction**	
Procedure	A pestle and mortar were pre-cooled by adding liquid nitrogen. Once nearly all evaporated, the leaf disc sample was added to the mortar and ground to a fine powder. 20-30 mg were then used for RNA extraction.
Kit	NucleoSpin™ RNA Plant Kit (Macherey-Nagel)
DNase treatment	On-column treatment included as part of the kit (above). Each column was treated with 95 µl DNase solution for 15 min at room temperature.
RNA assessment	Purity and yield were measured using an LVis plate with SpectroStar Nano microplate reader (BMG Labtech) by evaluating absorbance ratios at 260/280 and 260/230 nm. Extractions with yield > 140 ng/µl, a 260/280 ratio near 2.0 and a 260/230 ratio >1.8 were selected.
**Reverse transcription**	
Complete reaction conditions	A subsample of 1 µg RNA was added to a 10 µl reaction containing 0.5 µl oligo-dT and 0.5 µl random nonamer primers, incubated for 5 min at 65 °C and immediately cooled on ice. Buffer, dNTP's, nuclease-free water and nanoScript2™ enzyme were added according to the instructions in the Precision nanoScript™ Reverse Transcription Kit (Primer Design). The final 20 µl reaction was incubated for 20 min at 42 °C, then 10 min at 75 °C. All cDNA was stored at -20 °C and diluted 1:5 prior to running RT-qPCR.
**RT-qPCR target and oligonucleotide information**	
See Supplementary Table 4 for gene IDs, primer sequences and amplicon lengths.<br>Primers were manufactured by Integrated DNA Technologies™ (IDT) and purified by desalting.	
**RT-qPCR protocol**	
RT-qPCR conditions	Hot start 95 °C for 2 min, then 40 cycles at 95 °C for 15 s and 60 °C for 1 min.
Melt curve	95 °C for 1 min, 60 °C for 30 s and 95 °C for 30 s

Reaction volume	15 µl
cDNA amount	4 µl (40ng)
Master mix	PrecisionPLUS qPCR Master Mix (Primer Design)
Primer concentration	0.467 µM
RT-qPCR instrument	AriaMx Real-Time PCR System (Agilent)
**RT-qPCR validation**	
Specificity	Checked for single peak in melt curve.<br>PCR products run on a gel and sequenced.
Primer efficiency / slope / y-intercept / R^2 of linear regression of C_q versus ln(cDNA) / C_q for NTC / C_q for minus RT control	Primer efficiencies were calculated as described by Pfaffl (2001).<br>C_q threshold = 450<br>NA where C_q did not reach threshold
HSP20	2.06 / -3.19 / 17.97 / 0.9970 / NA / 37.60
Rca1β	1.98 / -3.37 / 13.95 / 0.9993 / NA / 28.25
Rca8α	1.97 / -3.39 / 16.77 / 0.9965 / NA / NA
Rca10α	1.91 / -3.57 / 18.61 / 0.9991 / NA / 30.33
Rca10β	2.03 / -3.25 / 23.95 / 0.9971 / NA / 28.47
Pp2A	2.01 / -3.30 / 21.75 / 0.9989 / NA / 27.17
Ubq28	1.95 / -3.44 / 19.80 / 0.9985 / NA / NA
PolyP	1.85 / -3.74 / 23.70 / 0.9992 / NA / NA
Elf1A	2.02 / -3.27 / 18.56 / 0.9934 / 37.67 / 31.83
B-Actin	1.95 / -3.45 / 23.22 / 0.9981 / NA / 30.30
Tua4	2.04 / -3.24 / 22.95 / 0.9963 / NA / 28.67
**Data analysis**	
Analysis software	AriaMx, version 2.1
Normalisation	The normalized relative quantity (NRQ) of expression was calculated in relation to the quantification cycle (C_q) values and the primer efficiency (E) of the target gene (goi) and the reference genes (ref1, ref2, ref3), based on Rieu & Powers (2009): $$NRQ = \frac{E_{goi}^{-Cq}}{\sqrt{E_{ref1}^{-Cq} \cdot E_{ref2}^{-Cq} \cdot E_{ref3}^{-Cq}}}$$
Number and justification of choice of reference genes	Six cowpea reference genes were selected from the literature (Da Silva *et al.*, 2015; Weiss *et al.*, 2018) and assessed for stability across 10 experimental samples that varied in terms of treatment (control and heat-treated samples) and leaf age. Both geNorm (Vandesompele *et al.*, 2002) (qbase+, Biogazelle) and Normfinder (Andersen *et al.*, 2004) software were used to analyse the results. The three most stable genes, Pp2a, PolyP and Ubi28, were used to normalise gene expression.
Statistical method	NRQ values were log transformed and evaluated for statistical significance using Two-Way ANOVA followed by post-hoc Tukey's test.

Table S8. RT-qPCR primers.

Type	Gene/transcript ID	Primer Name	Sequence (5' > 3')	Amplicon length (bp)
Control	Vigun06g055220	Vu06g052200_HSP20_F	GCTTCACCTAAAGTGCTGTTGA	147
		Vu06g052200_HSP20_R	CTTACTGTATTCATCGTCTCCTGC	
GOI	Vigun01g219300	VuRca1.1_F_v1	CTTGGAAATGCTAACGAAGATGC	156
		VuRca1.1_R_v2	CACAAGTGCCATCACAATTGC	
GOI	Vigun08g150700	VuRca8.1_F_v5	CTACCAGAGACGACCGAATTGG	128
		VuRca8.1_R_v5	CCTGAGTGCACCAAAGAAATCA	
GOI	Vigun10g051600.1	VuRca10.1_F_v2	GAAACTTCTATGGACAAGCAGCT	134
		VuRca10.1_R_v2	CATCACCTAAAGTGTGTATGTGCA	
GOI	Vigun10g051600.2	VuRca10.2_F_v1	TGCTTGTCCAAGAGCAAGAGA	144
		VuRca10.2_R_v1	ACAACTTTAGGATCAAATCAAGGGT	
Reference	Vigun07g271800	VuPp2A_F_v2	TCAGCATATTCTTCCTTGTGTGA	101
		VuPp2A_R_v2	CTAAGACTGGTGCCATTCCC	
Reference	Vigun06g121000	VuUbq28_F	GAGCTCAAGGACCTCCAGAA	130
		VuUbq28_F	CTAGAAAAACACCCCCAGCA	
Reference	Vigun06g003800	VuPolyP_F	CATGCAGACCACAAGGATTGA	172
		VuPolyP_R	GAGGAGGTGACTGGCACAT	
Reference	Vigun04g088900	VuElf1A_Fv_2	GCTGTAACAAAATGGATGCCAC	120
		VuElf1A_Rv_2	CAAATGGAATCTTGTCTGGGTTG	
Reference	Vigun04g203000	VuBAct_F_v2	CTTCCAGCAGATGTGGATTGC	146
		VuBAct_R_v2	GCAGGCAGCAGTTGTTCC	
Reference	Vigun03g291000	VuTua4_F_v2	GGCGTTCCCTTGACATTGAG	182
		VuTua4_R_v2	GGGAGCATAAGATGAAAGCATAAAG	

Table S9. Modelling of the temperature response of Rubisco activation by cowpea (*V. unguiculata* L.) Rca in leaf extracts of control and heat-treat plants. The model providing the best fit to the data is highlighted in bold and was selected according to the lowest AIC score (Akaike information criterion) calculated according to using the AIC function in R. Models were applied to the full dataset shown in Fig. 4 (n=6-8).

Treatment	Rubisco activation		
	Model	degrees of freedom (K)	AIC score
Control	2nd order poly	4	-96
	3rd order poly	5	-103
	**4th order poly**	**6**	**-103**
	5th order poly	7	-101
	GAM	3	-6
Heat	2nd order poly	4	-94
	3rd order poly	5	-112
	**4th order poly**	**6**	**-113**
	5th order poly	7	-111
	GAM	3	-77

Table S10. Leaf total soluble protein (TSP) and chlorophyll content of control and heat-treated cowpea (*V. unguiculata* L.) plants. Leaf extracts used were aliquoted from the Rca temperature response measurements. TSP (n= 55-56), total chlorophyll (n= 54), chlorophyll a/b (n=50-55). Values shown here are the mean ± standard error of the mean of values determined for individual biological replicates. All analysis conducted on the fifth day of heatwave. p-value and t-value determined from t-test (ns: $p > 0.05$, *: $p \leq 0.05$, **: $p \leq 0.01$, ***: $p \leq 0.001$).

Data Set	Treatment	Mean	p-value	t-value
TSP (µg/µl)	control	3.65 ± 0.45	0.002 (**)	3.163
	heat	3.35 ± 0.53		
Total chlorophyll (mg/m^2)	control	434 ± 42.3	0.455 (ns)	-0.750
	heat	439 ± 35.1		
Chlorophyll a/b	control	2.22 ± 0.04	0.000183 (***)	-3.886
	heat	2.26 ± 0.06		

References

Akaike H. **1974**. A new look at the statistical model identification. *IEEE Transactions on Automatic Control* **19**: 716–723.

Andersen CL, Jensen JL, Ørntoft TF. **2004**. Normalization of real-time quantitative reverse transcription-PCR data: a model-based variance estimation approach to identify genes suited for normalization, applied to bladder and colon cancer data sets. *Cancer Research* **64**: 5245–5250.

Bloemers D, Carmo-Silva E. **2024**. Antibody design for the quantification of photosynthetic proteins and their isoforms. In: Covshoff S, ed. Photosynthesis : Methods and Protocols. New York, NY: Springer US, 405–416.

Bustin SA, Benes V, Garson JA, Hellemans J, Huggett J, Kubista M, Mueller R, Nolan T, Pfaffl MW, Shipley GL, *et al.* **2009**. The MIQE Guidelines: Minimum information for publication of quantitative real-time PCR experiments. *Clinical Chemistry* **55**: 611–622.

Da Silva HAP, Nardeli SM, Alves-Ferreira M, Simões-Araújo JL. **2015**. Evaluation of reference genes for RT-qPCR normalization in cowpea under drought stress during biological nitrogen fixation. *Crop Science* **55**: 1660–1672.

Jung H-S, Crisp PA, Estavillo GM, Cole B, Hong F, Mockler TC, Pogson BJ, Chory J. **2013**. Subset of heat-shock transcription factors required for the early response of Arabidopsis to excess light. *Proceedings of the National Academy of Sciences* **110**: 14474–14479.

Pfaffl MW. **2001**. A new mathematical model for relative quantification in real-time RT–PCR. *Nucleic Acids Research* **29**: e45.

Rieu I, Powers SJ. **2009**. Real-time quantitative RT-PCR: design, calculations, and statistics. *The Plant Cell* **21**: 1031.

Stotz M, Mueller-Cajar O, Ciniawsky S, Wendler P, Hartl FU, Bracher A, Hayer-Hartl M. **2011**. 1 3 6 6 VOLUME 18 NUMBER 12 DECEMBER 2011 nature structural & molecular biology. *Nature Structural & Molecular Biology*.

Vandesompele J, De Preter K, Pattyn F, Poppe B, Van Roy N, De Paepe A, Speleman F. **2002**. Accurate normalization of real-time quantitative RT-PCR data by geometric averaging of multiple internal control genes. *Genome Biology* **3**: research0034.1.

Weiss J, Terry MI, Martos-Fuentes M, Letourneux L, Ruiz-Hernández V, Fernández JA, Egea-Cortines M. **2018**. Diel pattern of circadian clock and storage protein gene expression in leaves and during seed filling in cowpea (Vigna unguiculata). *BMC Plant Biology* **18**: 33.
